# Acquired immunostimulatory phenotype of migratory CD103^+^ DCs promotes alloimmunity following corneal transplantation

**DOI:** 10.1172/jci.insight.182469

**Published:** 2024-10-22

**Authors:** Tomás Blanco, Hayate Nakagawa, Aytan Musayeva, Mark Krauthammer, Rohan Bir Singh, Akitomo Narimatsu, Hongyan Ge, Sara I. Shoushtari, Reza Dana

**Affiliations:** Laboratory of Ocular Immunology, Transplantation and Regeneration, Schepens Eye Research Institute of Massachusetts Eye and Ear, Department of Ophthalmology, Harvard Medical School, Boston, Massachusetts, USA.

**Keywords:** Immunology, Ophthalmology, Dendritic cells, Organ transplantation, Tolerance

## Abstract

After transplantation, Th1-mediated immune rejection is the predominant cause of graft failure. Th1 cell sensitization occurs through complex and context-dependent interaction among antigen-presenting cell subsets, particularly CD11b^+^ DCs (DC2) and CD103^+^ DCs (DC1). This interaction necessitates further investigation in the context of transplant immunity. We used well-established preclinical models of corneal transplantation and identified distinct roles of migratory CD103^+^ DC1 in influencing the outcomes of the grafted tissue. In recipients with uninflamed corneal beds, migratory CD103^+^ DC1 demonstrate a tolerogenic phenotype that modulates the immunogenic capacity of CD11b^+^ DC2 primarily mediated by IL-10, suppressing alloreactive CD4^+^ Th1 cells via the PD-L1/PD-1 pathway and enhancing Treg-mediated tolerance via αvβ8 integrin–activated TGF-β1, thus facilitating graft survival. Conversely, in recipients with inflamed and vascularized corneal beds, IFN-γ produced by CD4^+^ Th1 cells induced migratory CD103^+^ DC1 to adopt an immunostimulatory phenotype, characterized by the downregulation of regulatory markers, including αvβ8 integrin and IL-10, and the upregulation of IL-12 and costimulatory molecules CD80/86, resulting in graft failure. The adoptive transfer of ex vivo induced tolerogenic CD103^+^ DC1 (iDC1) effectively inhibited Th1 polarization and preserved the tolerogenic phenotype of their physiological counterparts. Collectively, our findings underscore the essential role played by CD103^+^ DC1 in modulating host alloimmune responses.

## Abstract 2

Using a consolidated preclinical model of corneal transplantation, we shed light on the graft immune rejection mechanisms, the leading cause of transplant failure

## Graphical abstract

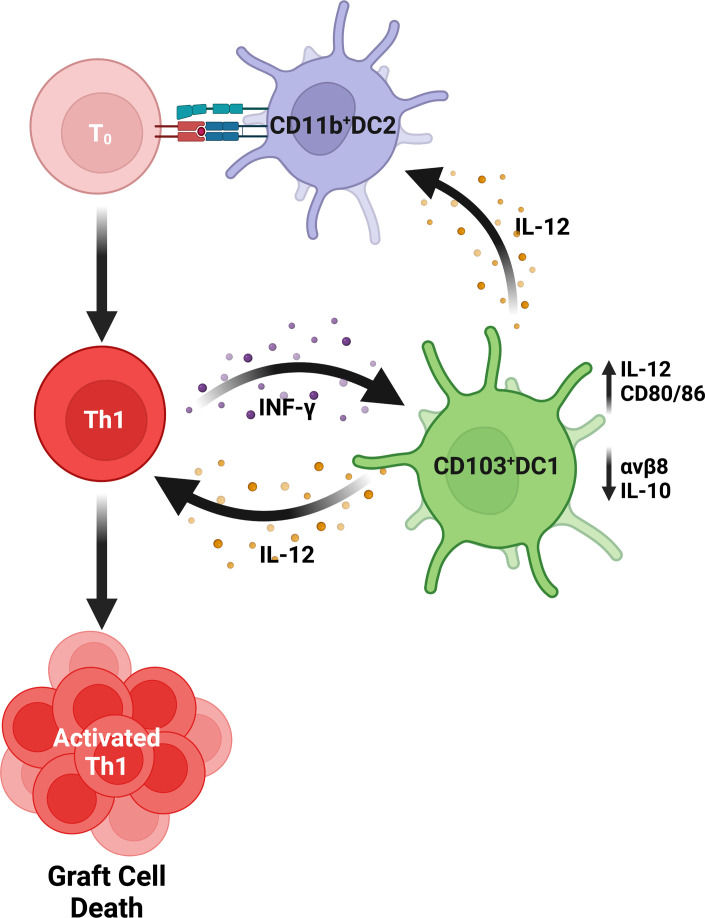

## Introduction

Corneal transplantation is the most common form of solid tissue grafting; however, the high success rate is overshadowed by the inherent reactivity of the immune system toward allogeneic tissues (1–3). Immune-mediated rejection of the transplanted corneal tissue is the most common cause for graft failure following penetrating keratoplasty, and ~30% of transplanted corneas undergo at least 1 episode of immune-mediated rejection, eventually leading to graft failure (4). Immunosuppressive drug therapies have been proven to be efficacious in dampening the host’s immunological responses (5, 6); however, their long-term use can cause systemic and local adverse effects such as glaucoma and cataracts or opportunistic infections (7, 8).

The current evidence suggests the presence of distinct phenotypic immune-quiescent myeloid cells in the normal cornea, including Langerhans cells, type 2 conventional CD11b^+^ DCs (DC2), macrophages, and plasmacytoid DCs (pDC) (9–13). These cells play a critical role in linking innate and adaptive immune responses when the corneal integrity is compromised (14–18). We have previously investigated the role of donor and host CD11b^+^ DC2 as the primary subset in driving corneal transplant immunity, by orchestrating generation of Th1 effector cells that are responsible for graft failure, especially in hosts with inflamed corneal beds in high risk (HR) setting with high rejection rates. In contrast, recipients with noninflamed corneal beds in low risk (LR) have a significantly lower rejection rates that are associated with poor capability of CD11b^+^ DC2 to orchestrate a potent Th1-mediated immune response (3, 18, 19).

Recently, we uncovered a potentially novel regulatory function for host CD103^+^ DC1 (profiled by the lineage markers IRF8, ID2, BATF3, XCR1, and CD370 but not IRF4, SIRP-α, CSFR, or CX3CR1) in promoting graft survival in LR recipients (20). These cells induce Treg-mediated tolerance to the graft concomitant with their expression of regulatory markers such as BTLA, αvβ8, ALDH, and PD-L1 (20).

Graft tissue–derived CD103^+^ DC1 are the most potent antigen-presenting cells (APC) that sensitize naive CD8^+^ T cells in the host by antigen cross-presentation promoting transplant immunity in other types of solid organ transplantation. However, the lack of CD103^+^ DC1 in the donor cornea (20) refutes the paradigm of the direct pathway of sensitization by this subset. Moreover, the corneal graft failure is primarily mediated by CD4^+^ (and not CD8^+^ ) Th1 cells (21). Therefore, it is relevant to delineate the role of the host’s migratory CD103^+^ DC1 following corneal transplantation, especially in recipients with grafts at HR of rejection.

Herein, we use well-established LR and HR graft recipient models of corneal transplantation to delineate the differential function phenotype of migratory CD103^+^ DC1, depending on the graft environment. In LR graft recipients, CD103^+^ DC1 show a tolerogenic phenotype associated with their capacity to phagocytize apoptotic cells. On the contrary, these cells acquire an immunostimulatory phenotype in HR graft recipients, after sensing IFN-γ secreted by alloreactive CD4^+^ Th1 cells in the draining lymph node (DLN). We profile this acquired immunostimulatory phenotype by the upregulated expression of IL-12, costimulatory CD80/86 molecules, and downregulated IL-10. Moreover, we identify, for the first time to our knowledge, a role of αvβ8 integrin in allograft tolerance, expressed by this subset.

## Results

### CD103^+^ DC1 infiltrate the graft site following corneal transplantation.

**Figure 1:**
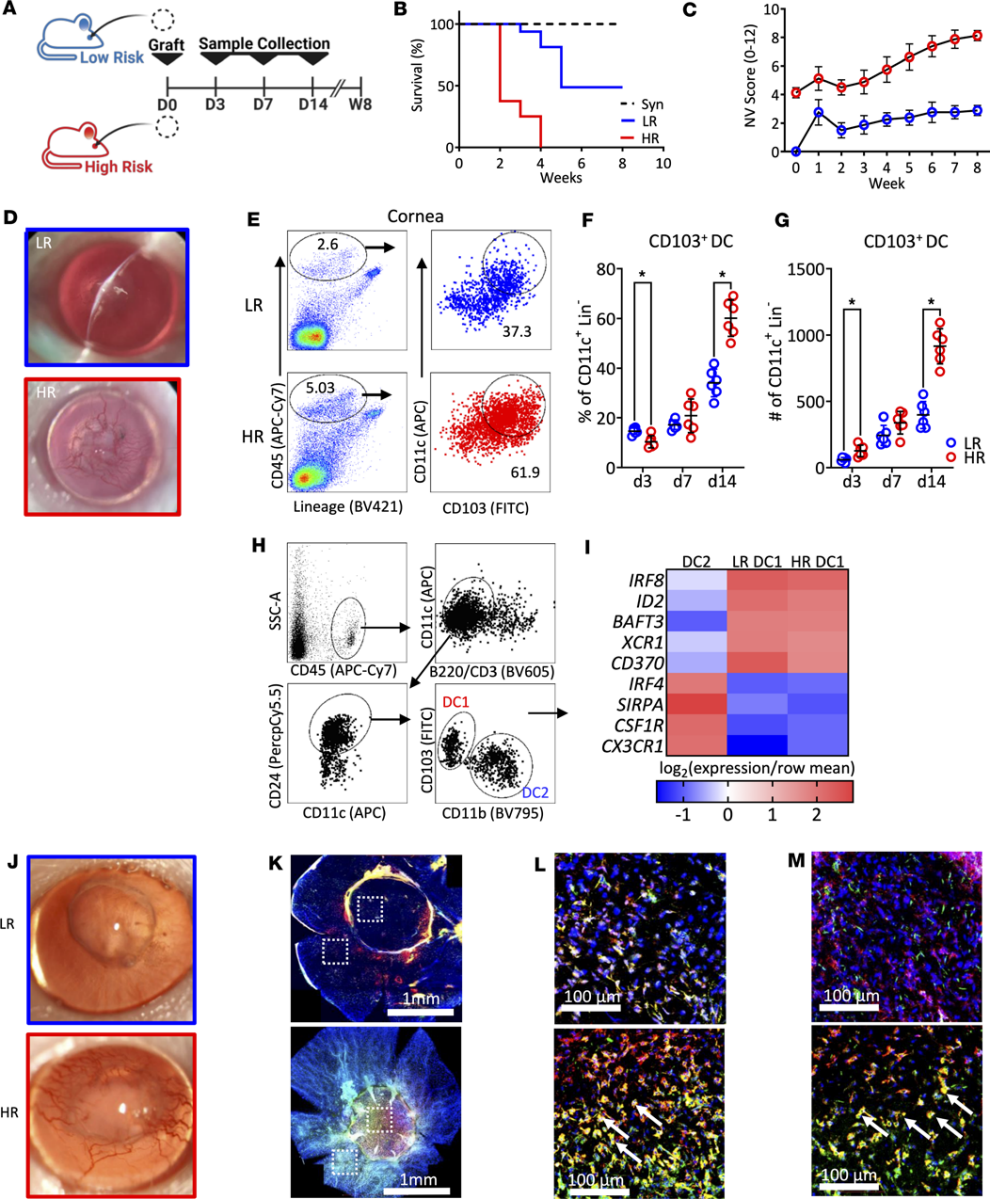
DC1 infiltration at the graft site. (**A**) Experimental schematic. Allogeneic (C57BL/6J) or syngeneic (BALB/C) corneal grafts were transplanted into HR or LR BALB/C recipient mice (*n* = 8/group). Mice were followed up for 8 weeks by slit lamp examination. (**B**) Graft survival was determined by opacification score (score: 0–5). (**C**) Neovascularization (NV) score (score: 0–8). Additionally, graft recipients (*n* = 6/group/time point) were euthanized on days 3, 7, and 14, and the grafted corneas were harvested, digested, and analyzed by flow cytometry. (**D**) Representative slit lamp images of accepted graft (LR) upper part, and rejected graft (HR) lower part. (**E**–**G**) The gate on CD103^+^CD11c^+^ was set for CD103^+^ DC. Mean (± SEM) of frequencies and numbers of CD103^+^ DC in the grafted corneas were measured. (**H**) Gating strategy for sorting CD103^+^ DC and CD11b^+^ DC from the grafted cornea following previous gating (Supplemental Figure 1). Gate B220^–^ versus CD11c^+^ was set to exclude pDC. The gate on CD11c^+^CD24^+^ cells was set to exclude contaminating macrophages/monocytes. The gate on CD11b^–^CD103^+^ cells was set for CD103^+^ DC. The gate on CD11b^+^CD103^–^ cells was set for CD11b^+^ DC. (**I**) Transcriptional programs expressed by DC subsets sorted by FACS were analyzed by qPCR assay, showing expression average of most differentially in CD103^+^ DC1 (relative to their expression in CD11b^+^ DC2 from pooled allografted corneas; *n* = 6 mice/group). (**J**) Representative slit lamp photography of LR and HR grafted corneas at 2 weeks after transplantation. The grafted corneas were excised, incubated in 20 nM EDTA to remove the epithelium, fixed with ice-cold acetone (*n* = 5), and stained. (**K**) Representative immunofluorescence of CD11c^+^ cells (red), CD11c^+^CD103^+^ (white arrows) represents CD103^+^ DC1, and single-positive (green) represents other CD103^+^ subsets (Tregs). (**L** and **M**) Representative sections of the host bed (**L**) and donor graft (**M**). Each symbol (**F** and **G**) indicates an individual mouse. *P < 0.05 (2-tailed *t* test). LR, low risk; HR, high risk. Scale bars: 1 mm (**K**), 100 µm (**L** and **M**). All results are of 1 experiment, with no repetitions in animal numbers.

Since CD103^+^ DC1 are scarce in the normal cornea (20), we first quantified the infiltration of these cells into the graft site (grafted cornea and recipient conjunctiva). We used 2 different validated mouse models of penetrant keratoplasty: (a) grafts transplanted to healthy corneal beds that are at LR of rejection and (b) grafts transplanted to inflamed vascularized corneal beds that are at HR of rejection (22). Animals were followed for 8 weeks (Figure 1A). While grafts enjoyed survival in ~50% of the LR recipients, immune rejection (verified in vivo by both endothelial failure and graft opacity) was universally observed in HR recipients (Figure 1B) with significantly higher neovascularization scores (Figure 1, C and D). At different time points, both grafted corneas and conjunctivae were harvested separately and assessed by flow cytometry (gating strategy in Supplemental Figure 1C; supplemental material available online with this article; https://doi.org/10.1172/jci.insight.182469DS1). Infiltration of CD103^+^ DC was observed in both LR and HR recipients (gating strategy in Supplemental Figure 1D); however, their frequencies were higher in HR grafted corneas (Figure 1, E–G) and conjunctivae (Supplemental Figure 1, F and H). We observed similar kinetics of CD11b^+^ DC2 (profiled as CD45^+^MHC-II^hi^CD11b^+^ DC2) cells (Supplemental Figure 1, J–L).

Next, we sorted CD103^+^ DC and CD11b^+^ DC separately from the grafted cornea or the conjunctiva (Figure 1H). The quantitative PCR (qPCR) analysis of CD103^+^ DC1 showed similar expression levels of prototypical lineage markers such as *IRF8*, *ID2*, *BATF3*, *XCR1*, and *CD370* but variable expression levels of *IRF4*, *SIRPA*, *CSFR*, and *CX3CR1* (distinguishing these cells from CD11b^+^ DC2) in the grafted corneas (Figure 1I) and conjunctivae (Supplemental Figure 1I) in HR and LR recipients. Moreover, immunofluorescence assays showed increased infiltration of CD103^+^ DC1 in donor grafts in HR compared with LR recipients (Figure 1, J–M). These data indicate higher CD103^+^ DC1 infiltration into the graft site following corneal transplantation in HR compared with LR recipients, providing evidence for this subset’s role in the host immune response.

### Migratory CD103^+^ DC1 acquire an immunostimulatory phenotype in HR transplantation.

The host immune response following corneal transplantation has been attributed to CD11b^+^ DC (3, 18). This subset showed higher frequencies (profiled as CD45^+^B220^–^CD11c^+^CD11b^+^MHCII^hi^; gating strategy Supplemental Figure 2 A, C, and F) in HR recipients (Supplemental Figure 2, G and H). The increased frequencies of CD11b^+^ DC correlate with the elevated IFN-γ^+^ alloreactive CD4^+^ T cells (Supplemental Figure 2, I–K) and graft failure (Figure 1B). However, some aspects such as the generation of spontaneous tolerance in LR recipients or the enhanced alloreactivity observed in HR recipients (3) cannot be explained by differential infiltration of CD11b^+^ DC cells observed in HR and LR models.

**Figure 2:**
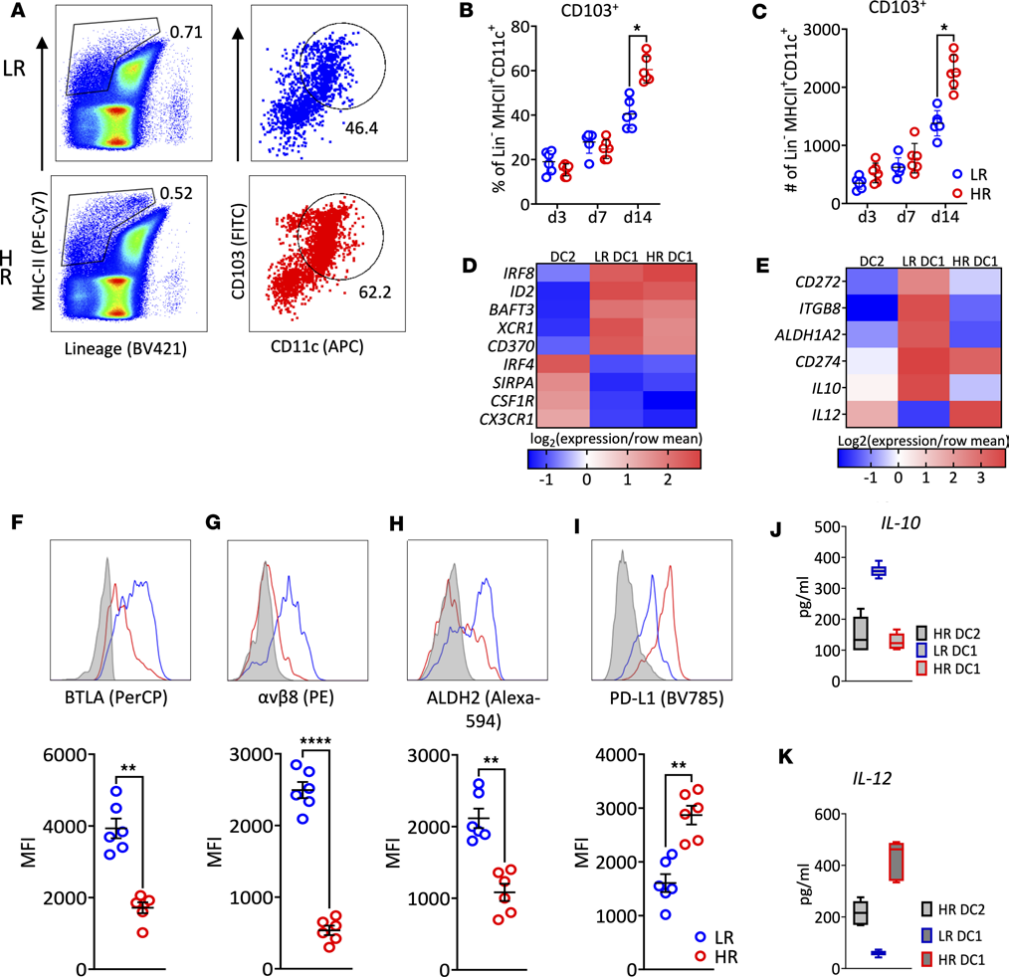
Migratory CD103^+^ DC1 in the DLN. DLN from graft recipients (*n* = 6/group/time point; same mice as from Figure 1) were analyzed by flow cytometry (gating strategy for evaluating CD103^+^ DC1 in the DLN shown in Supplemental Figure 2). (**A**) Gate on lineage^–^MHC-II^+^ was set to include mature lineage^–^ cells, and the gate on CD103^+^CD11c^+^ cells was set for frequencies of DC1. (**B** and **C**) Mean (± SEM) of frequency (**B**) and numbers (**C**) of CD103^+^CD11b^–^ in the ipsilateral DLN of graft recipient mice were measured (gating strategy for sorting DC1 from DC2 in the DLN at 2 weeks after transplant shown in Supplemental Figure 2, A and B). (**D** and **E**) Transcriptional programs and markers expressed by DC subsets sorted by FACS from the DLN of allograft recipient mice at 2 weeks after transplant were analyzed by qPCR assay and show expression average of most differentially in DC1 relative to their expression in DC2 from pooled DLN (*n* = 6 mice/group, same mice as from Figure 1; gating strategy for evaluating DC1 and DC2 phenotypic markers in the DLN shown in Supplemental Figure 2, A and B). (**F**–**I**) The median fluorescence intensity (MFI) of regulatory markers was measured and compared in DC1 form either LR or HR recipients at 2 weeks after transplant (*n* = 6/group, gating from above). Each symbol in **F**–**I** indicates an individual mouse. Additionally, sorted DC1 and DC2 were cultured and challenged with irradiated apoptotic corneal cells, stimulated with PMI and ionomycin. (**J** and **K**) ELISA assessed the supernatant for IL-10 (**J**) and IL-12 (**K**) secretion. Results are of 2 sets of triplicates with cells pooled from 3 mice on each set. *P < 0.05, **P < 0.01, ****P < 0.0001 (2-tailed *t* test). All results are of 1 experiment with no repetitions in animal numbers.

The role of CD103^+^ DC1 in orchestrating the immune response in other forms of solid organ transplantation is well characterized (23–25). Therefore, we first analyzed migratory CD103^+^ DC (profiled as CD45^+^Lin^–^MHC-II^hi^Cd11c^+^CD103^+^) from the DLN (Figure 2A and Supplemental Figure 2, A–E) and found significantly higher frequencies in HR recipients (Figure 2, B and C). Moreover, the gene expression levels of CD103^+^ DC lineage markers were similarly conserved in these cells isolated from the graft side of HR and LR (Figure 2D) recipients (Figure 1I and Supplemental Figure 1I).

We next examined the expression levels of regulatory markers in CD103^+^ DC1. Migratory CD103^+^ DC1 from HR recipients showed downregulated BTLA, αvβ8, ALDH2, and IL-10 and upregulated PD-L1 (Figure 2, E–J), displaying an immunostimulatory profile with high expression of CD80/86 and IL-12 (Supplemental Figure 2, L and M). These data show for the first time to our knowledge that migratory CD103^+^ DC1 acquire an immunostimulatory phenotype in HR recipients following corneal transplantation, independent of their lineage signature.

### CD103^+^ DC1 immunostimulatory phenotype is induced by IFN-γ secreted by alloreactive Th1 CD4^+^ T cells.

**Figure 3:**
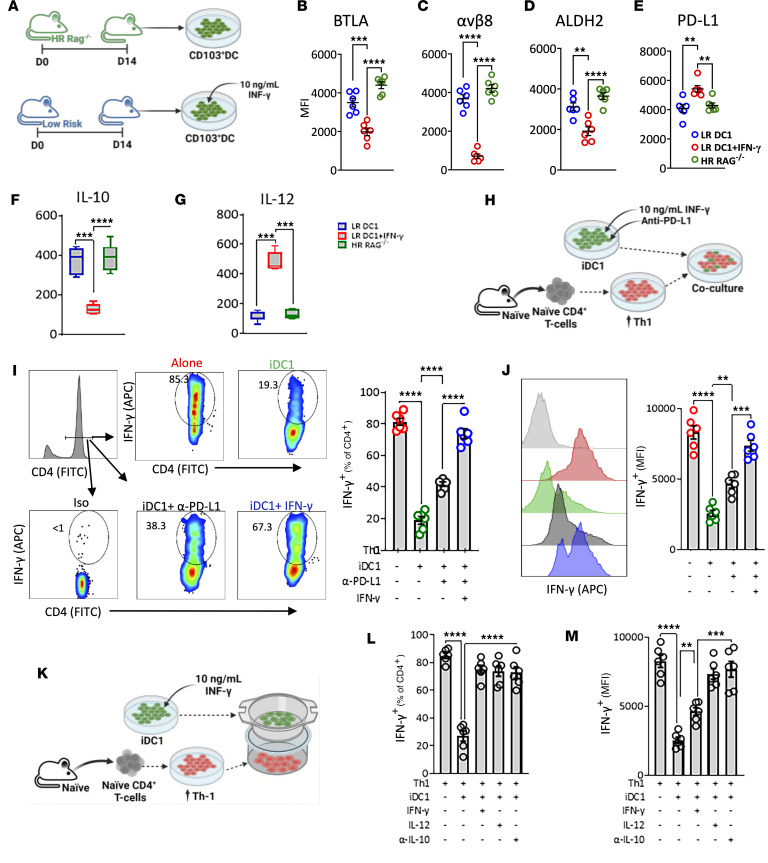
Immunostimulatory profile of migratory CD103 DC1. (**A**) Experimental schematic. Allogeneic (C57BL/6J) corneal grafts were transplanted into BALB/C LR and HR RAG^–/–^ recipient mice. Two weeks after transplantation, CD103^+^ DC1 were sorted from the migratory compartment, as described above. CD103^+^ DC1 from LR recipients were incubated with IFN-γ, and those from HR RAG^–/–^ were left untreated. (**B**–**E**, **F**, and **G**) After 12 hours, cells were analyzed by flow cytometry for the expression of regulatory markers (**B**–**E**) and supernatant for the secretion of IL-10 (**F**) and IL-12 (**G**). Each symbol in **B**–**E** indicates an individual mouse. (**H**) iDC1 (Supplemental Figure 2) were sorted by FACS and preincubated with or without IFN-γ and then cocultured with in vitro–differentiated Th1 CD4^+^ T cells with or without anti–PD-L1 blocking antibodies. (**I** and **J**) The intracellular expression of IFN-γ by CD4^+^ T cells was analyzed by flow cytometry, and the mean (± SEM) of frequencies (**I**) and expression (MFI) (**J**) were measured. Results show 2 sets of triplicates. (**K**) iDC1 (Supplemental Figure 3, A and B) were sorted by FACS and preincubated with or without IFN-γ. iDC1 were transwell plated (upper) with in vitro–differentiated Th1 CD4^+^ T cells (lower). Additionally, mouse recombinant IL-12 or anti-IL-10 were added. (**L** and **M**) The intracellular expression of IFN-γ by CD4^+^ T cells was analyzed by flow cytometry, and the mean (± SEM) of frequency (**L**) and expression (MFI) (**M**) was measured. Results show 2 sets of triplicates. **P < 0.01, ***P < 0.001, ****P < 0.0001 (1-way ANOVA with Bonferroni correction). All results are of one experiment with no repetitions. Experimental schematics created with Biorender.com.

Next, we explored the underlying mechanisms that could induce CD103^+^ DC1 reprogramming. The IFN-γ expression by CD11b^+^ DC2–sensitized alloreactive CD4^+^ T cells is a fundamental feature of HR graft recipients (18, 19) that is also observed downstream in the DLN (Supplemental Figure 2, H–J). IFN-γ is a primary driver in the induction of the CD103^+^ DC1 immunostimulatory phenotype at baseline or upon antigen uptake in vivo (26, 27); we next investigated this following corneal transplantation. Migratory CD103^+^ DC1 were sorted (gating strategy Supplemental Figure 2E) and incubated with IFN-γ (Figure 3A). In the previous experiment, we observed decreased expression levels of regulatory markers and upregulated PD-L1 in HR recipients. Additionally, low IL-10 and high IL-12 protein levels were observed in HR recipients. On the contrary, in HR RAG^–/–^ recipients, migratory CD103^+^ DC1 showed a tolerogenic profile (Figure 3, B–G), similar to their LR recipient counterparts (Figure 2, F–K).

Next, we generated tolerogenic iDC1 ex vivo (described in ref. 18). DC1 were challenged with allogeneic ultraviolet-irradiated apoptotic corneal cells (Supplemental Figure 3, A and B). Subsequently, these cells underwent maturation with high expression levels of MHC-II and CCR7 (Supplemental Figure 3, C and D). The differentiated iDC1 showed upregulated αvβ8, PD-L1, and IL-10 and low expression of IL-12 (Supplemental Figure 3, E–H) similar to their physiologic counterparts in LR recipients (Figure 2, F–K). However, the treatment of iDC1 with IFN-γ induced an immunostimulatory phenotype (Supplemental Figure 3, E–H) similar to their physiological HR counterparts (Figure 2, F–K).

We assessed the capacity of iDC1 to inhibit ex vivo–polarized Th1 IFN-γ–producing cells (Figure 3H). iDC1 potently inhibited IFN-γ^+^CD4^+^ T cells; however, addition of either anti–PD-L1 blocking antibodies or pretreatment with IFN-γ subverted such capacity (Figure 3, I and J). Interestingly, we observed that IFN-γ–induced loss of iDC1 inhibitory capacity was more than checkpoint inhibition in both CD103^+^ DC1. Additionally, iDC1 treated with IFN-γ showed upregulated expression of PD-L1 (Supplemental Figure 3F).

Subsequently, we assessed the capacity of iDC1 to inhibit IFN-γ^+^CD4^+^ T cells in a transwell system (Figure 3K). We observed that iDC1 showed an independent PD-L1/PD-1 inhibitory capacity that was subverted when iDC1 were pretreated with IFN-γ (Figure 3, L and M). Since the IL-10/IL-12 ratio was lower in migratory CD103^+^ DC1 from HR recipients (Figure 2, E, J, and K) as well as in iDC1 treated with IFN-γ (Supplemental Figure 3, G and H), we assessed the role of these antagonistic cytokines. Adding either anti–IL-12 or anti–IL-10 antibodies revoked the inhibitory capacity of iDC1 (Figure 3, L and M). Collectively, these data indicate that tolerogenic CD103^+^ DC1 self-restrain their immunostimulatory function and control the threshold of alloreactive Th1 cells; however, IFN-γ led them to lose their inhibitory capacity and acquire an immunostimulatory phenotype.

### Migratory CD103^+^ DC1 fail in priming alloreactive CD4^+^ Th1 cells but enhance CD11b^+^ DC1 immunogenic capacity in HR recipients.

**Figure 4:**
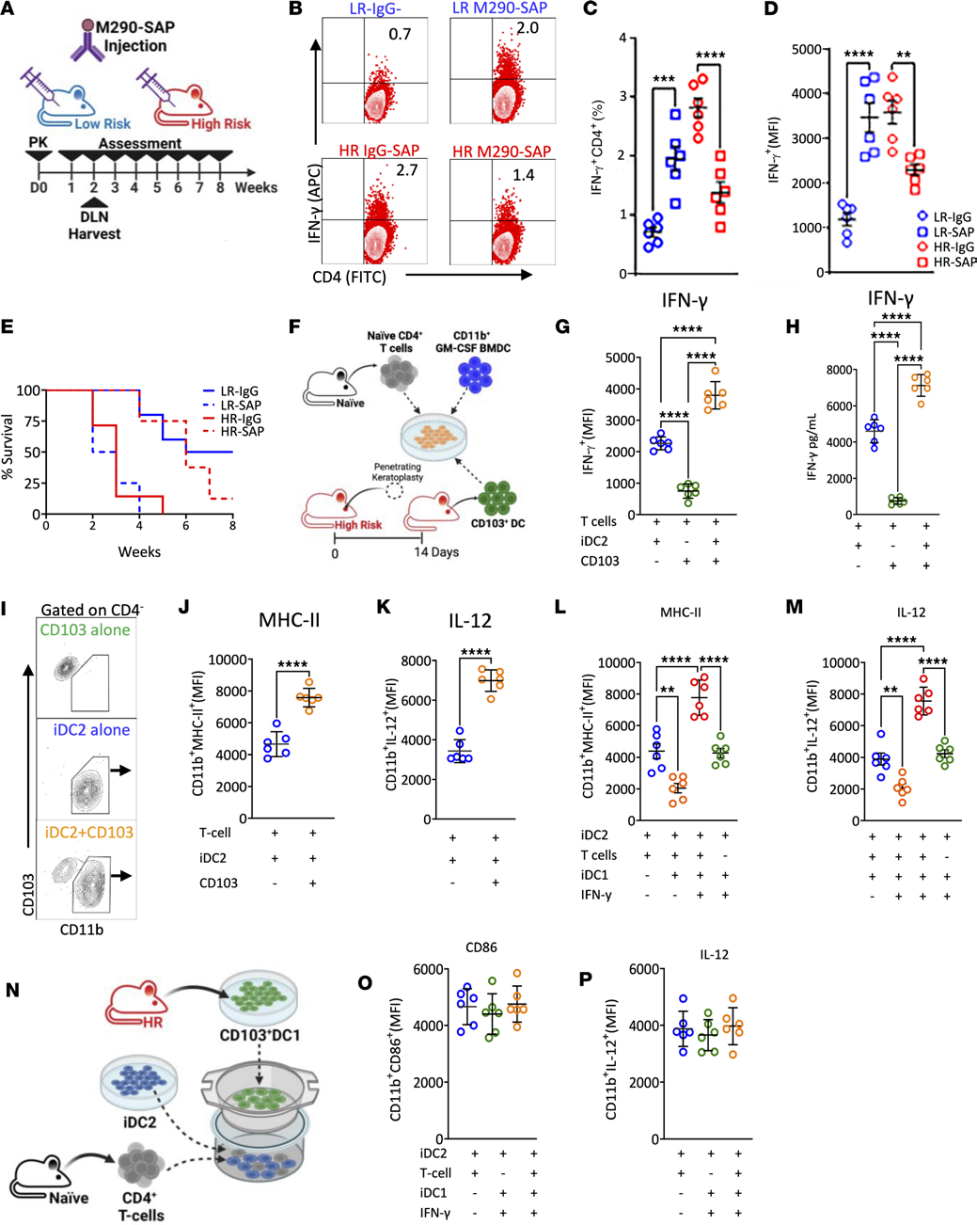
The effect of migratory CD103^+^ DC1 on corneal transplant immunity. (**A**) BALB/C LR or HR recipients were transplanted as above. Allograft recipient mice were then randomized; 1 group was i.p. injected with anti–M290-SAP, while the other group was treated with anti–IgG-SAP. (**B**–**D**) Two weeks after transplantation, mice were euthanized (*n* = 6/group), the intracellular expression of IFN-γ by CD4^+^ T cells from the DLN was analyzed by flow cytometry (**B**), frequency (**C**), and expression (MFI) (**D**) were measured. (**E**) Additional mice were followed(*n* = 8/group) for 8 weeks to evaluate graft opacity, and Kaplan-Meier curves were plotted to evaluate graft survival. (**F**) Two weeks after transplantation, CD103^+^ DC1 were sorted by FACS from the migratory compartment of the DLN of HR recipient mice. BMDC-derived CD11b^+^ DC2 (iDC2) were generated with GM-CSF and stimulated with LPS. Naive CD4^+^CD25^–^ T cells were sorted by MACS from the spleen of naive BALB/C mice. (**G** and **H**) Cells were cocultured for 3 days, and intracellular expression of IFN-γ by CD4^+^ T cells was analyzed by flow cytometry, ELISA, and MFI, and protein concentration in the supernatant was measured. (**I**–**K**) Gated CD4^–^CD11b^+^ DC2 were separated from CD103^+^ DC1 and analyzed by flow cytometry for MHC-II and IL-12 expression. Similar MLR was performed with iDC1 pretreated with or without IFN-γ. (**L** and **M**) CD4^–^CD11b^+^ DC2 were separated from CD103^+^ DC1 and analyzed by flow cytometry for MHC-II (**L**) and IL-12 (**M**) expression. (**N**) iDC2 and naive CD4^+^CD25^–^ T cells were cocultured in the lower and iDC1 pretreated with or without IFN-γ were plated in the upper chamber of transwell system. (**O** and **P**) Gates on CD4^–^CD11b^+^ DC2 were separated from CD103^+^ DC1 and analyzed by flow cytometry for CD86 (**O**) and IL-12 (**P**) expression. Results are of 2 sets of triplicates with cells pooled from 3 mice on each set. Plots represent the mean(± SEM). **P < 0.01, ****P < 0.0001 (2-tailed *t* test [**C**, **D**, **J**, and **K**] or 1-way ANOVA [**G**, **H**, **L**, **M**, **O**, **P**] with Bonferroni correction).

To further delineate the role of CD103^+^ DC1 in host immune response after transplantation, we depleted this subset by subconjunctival (SC) injection of anti–M290-SAP antibodies (Figure 4A) (as described in ref. 18). While depletion of CD103^+^ DC1 led to increased frequency of IFN-γ^+^ cells in the DLN of LR recipients, it decreased in HR recipients compared with IgG controls (Figure 4, B–D). The changes in frequencies of IFN-γ^+^ cells were also reflected in the changes in graft survival rates (Figure 4E).

To explore the direct contribution of migratory CD103^+^ DC1 to allosensitization, we set up a mixed lymphocyte reaction (MLR) of syngeneic CD4^+^CD25^–^ T cells with (a) mature iDC2 (positive control), (b) HR migratory CD103^+^ DC1 sorted by FACS, and (c) both iDC2 and migratory CD103^+^ DC1 (Figure 4F). We observed very low expression levels of IFN-γ by CD4^+^ T cells in migratory CD103^+^ DC1 compared with iDC2, but it was drastically increased when iDC2 and migratory CD103^+^ DC1 were cultured together (Figure 4, G and H). Moreover, in the presence of HR migratory CD103^+^ DC1, iDC2, profiled as CD11b^+^CD103^–^ (Figure 4I), showed an enhanced expression of immunostimulatory phenotype, profiled by increased MHC-II (Figure 4J) and IL-12 (Figure 4K), increased CD80/86, and decreased IL-10 (Supplemental Figure 4, A–C) levels. Coculturing iDC1 (preincubated with IFN-γ) with iDC2 with or without CD4^+^CD25^–^ T cells showed enhanced iDC2 immunostimulatory phenotype only in the presence of T cells (Figure 4, L and M). Finally, we performed a transwell coculture of CD4^+^CD25^–^ T cells with iDC2 (culture plate) with iDC1 pretreated with IFN-γ (transwell insert) (Figure 4N) and observed that the iDC2 phenotype remained unchanged (Figure 4, O and P). These data indicate that, in HR recipients, CD103^+^ DC1 enhance CD11b^+^ DC2 immunogenic capacity, which depends on T cell interaction.

### Migratory CD103^+^ DC1 fail to induce Treg-mediated allograft tolerance in HR recipients.

**Figure 5:**
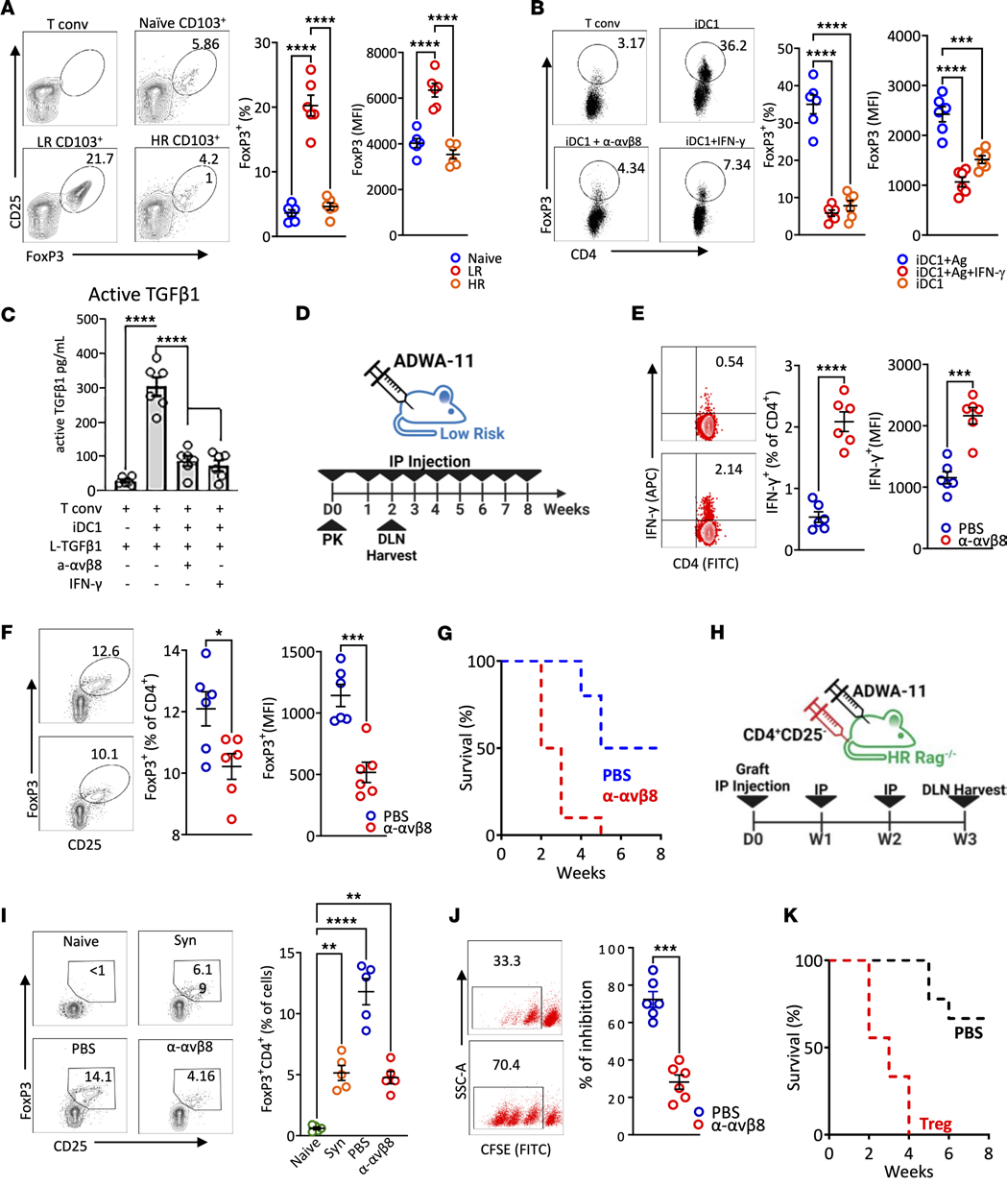
The effect of migratory CD103^+^ DC1 on tolerance. Migratory CD103^+^ DC1 from either LR or HR recipients were sorted as above, synchronized by starvation, and then cocultured with CD4^+^CD25^–^ T cells harvested from BABL/C naive mice. (**A**) On day 5, cells were harvested, and expression of FoxP3 in CD4^+^T cells was analyzed by flow cytometry. (**B**) iDC1 were treated with or without IFN-γ and were then synchronized by starvation and cultured with CD4^+^CD25^–^ T cells in SFM supplemented with latent-TGF-β1, IL-2, and with or without anti-αvβ8 blocking antibody. (**B** and **C**) On day 5, cells were harvested and the expression of FoxP3 in CD4^+^T cells was analyzed (**B**), and soluble active TGF-β1 in the supernatant was assessed (**C**). (**D**) LR graft recipient mice were treated with ADWA-11 antibody or IgG-control on the day of the transplantation and once a week. (**E** and **F**) Two weeks after transplantation, DLN CD4^+^ T cell intracellular expression of IFN-γ (**E**) and FoxP3 (**F**) was analyzed. (**G**) Additional mice were followed (*n* = 8/group) for 8 weeks through slit lamp examination to evaluate graft opacity, and graft survival was evaluated. (**H**) Syngeneic naive CD4^+^CD25^–^ T cells were transferred i.v. at the time of the transplantation into HR Rag^–/–^ recipient mice. Mice were treated with ADWA-11 antibody or IgG control on the day of the transplant and once a week. (**I** and **J**) On day 21 after transplantation, DLN were harvested, and expression of FoxP3 in CD4^+^ T cells (**I**) and their suppressive capacity in proliferation CFSE-labeled CD4^+^ T cell (**J**) was analyzed by flow cytometry. Tregs from the DLN of mice treated with IgG control were sorted by FACS and adopted into newly transplanted HR recipient mice, and control mice received PBS (*n* = 8/group). (**K**) Mice were followed for 8 weeks for evaluating graft opacity and graft survival. Results (**B** and **C**) are of 2 sets of triplicates. Plots represent the mean (± SEM). *P < 0.05, **P < 0.01, ***P < 0.001, ****P < 0.0001 (2-tailed *t* test [**E**, **F**, and **J**] or 1-way ANOVA [**A**, **B**, **C**, and **I**] with Bonferroni correction).

Flow cytometry analysis of Tregs in HR recipients revealed impaired functionality profiled by reduced FoxP3 expression (Supplemental Figure 5A) and loss of suppressive capacity (Supplemental Figure 5B) consistent with universal graft failure. Since CD103^+^ DC1 are essential for inducing antigen-specific Treg-mediated tolerance (28–31), we depleted CD103^+^ in allograft recipients and observed impaired FoxP3 expression (Supplemental Figure 5C). Next, we assessed the capacity of migratory CD103^+^ DC1 sorted by FACS to induce FoxP3^+^ Treg ex vivo. CD103^+^ DC1 from HR recipients failed to induce Tregs compared with their LR counterparts (Figure 5A). Induction of peripheral Tregs depends on the extracellular activation of TGF-β1 by the integrin αvβ8 (32–35), which is uniquely expressed by CD103^+^ DC1 (29, 32).

We evaluated the capacity of iDC1 in induction of FoxP3^+^ Tregs in serum-free media (SFM) supplemented with LAP–TGF-β1. We observed that iDC1 successfully induced FoxP3^+^ expression (Figure 5B) and activated TGF-β1 (Figure 5C); however, neither anti-αvβ8 blocking antibodies nor pretreatment with IFN-γ reduced the iDC-mediated Treg generation. Notably, TGF-β1 expression on iDC1 was below the threshold of detection (Supplemental Figure 5D). To further evaluate the involvement of αvβ8 integrin in Treg-mediated tolerance, LR recipients were treated with ADWA-11 (Figure 5D). These mice showed increased Th1 alloreactive T cells (Figure 5E), impaired Treg function (Figure 5F), and immediate graft failure (Figure 5G).

We transferred syngeneic CD4^+^CD25^–^ T cells into HR RAG^–/–^ recipients that underwent treatment with ADWA-11 and a regular diet in a pathogen-free environment (Figure 5H). Flow cytometry showed FoxP3^+^ Tregs (Figure 5I) with high suppressive capacity (Figure 5J) in untreated mice; however, ADWA-11 reduced Treg induction. Finally, on transferring Tregs induced in RAG^–/–^ mice into newly transplanted WT HR recipients, we observed increased allograft survival (Figure 5K).These data confirm that migratory CD103^+^ DC1 are key mediators in promoting Treg-mediated tolerance following transplantation. Moreover, for the first time to our knowledge in any kind of solid organ transplantation, we show the critical role of αvβ8 integrin in promoting immune tolerance to the graft.

### Adoptive transfer of iDC1 leads to the promotion of tolerance and graft survival.

**Figure 6:**
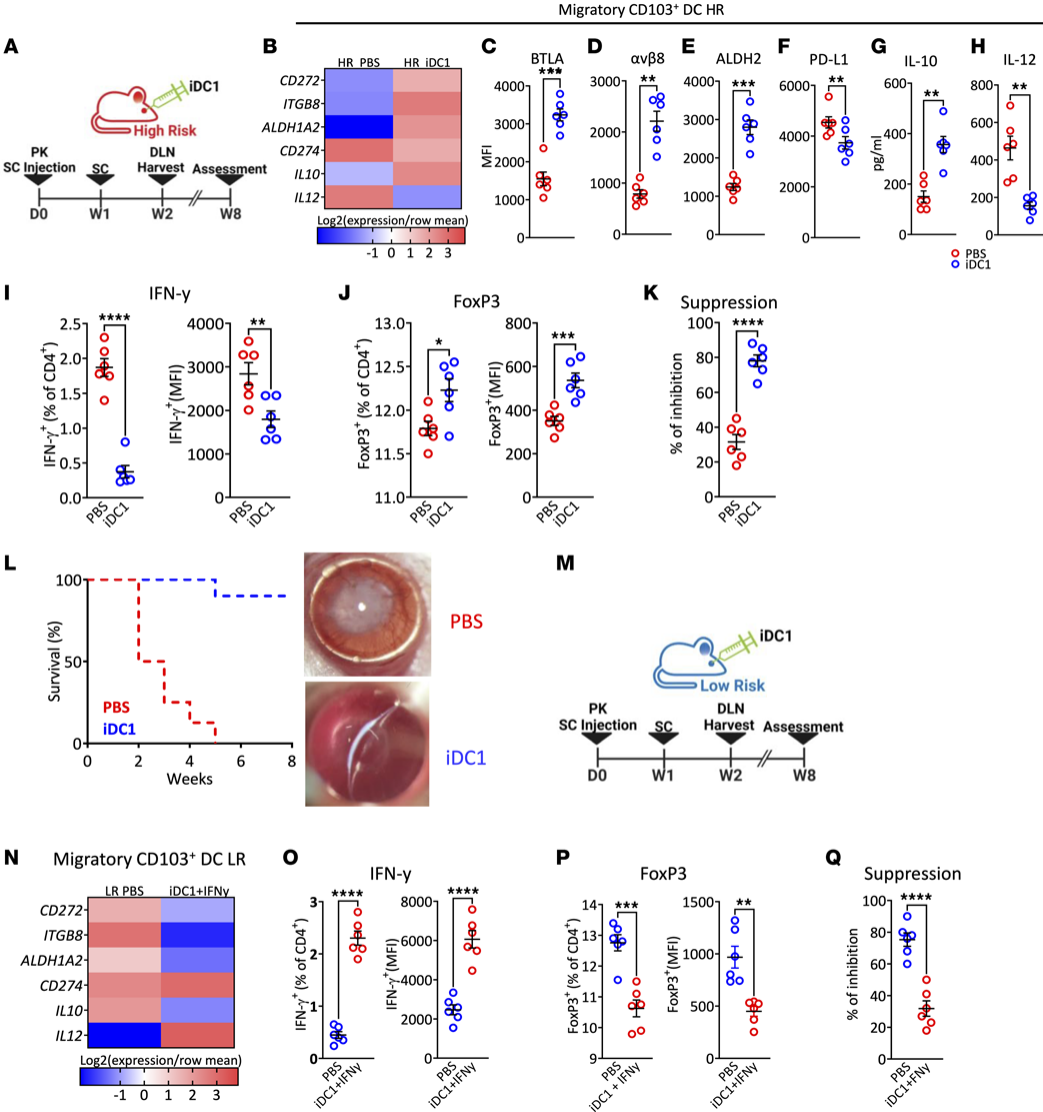
The effect of antigen-loaded iDC1 adoptively transferred in corneal transplant immunity. (**A**) BALB/C HR recipients were transplanted as above. Immediately after transplantation, allograft recipient mice received 2 × 10^4^ of CMTMR-labeled iDC1 or PBS via SC injection. (**B**) Two weeks after transplantation, CMTMR^–^ migratory CD103^+^ DC1 were sorted by FACS from the DLN and analyzed by qPCR assay, showing the expression average of most differentially functional regulatory markers. (**C**–**H**) The MFI of regulatory markers was measured by flow cytometry (**C**–**F**) or ELISA (**G** and **H**) and compared with DC2 from HR recipients (previous gating from above; Figure 1A). (**I** and **J**) Intracellular expression of IFN-γ by Th1 and FoxP3^+^CD25^+^ gated on CD3^+^CD4^+^ T cells from the DLN of graft recipient mice. (**K** and **L**) Ex vivo Treg suppressive capacity on proliferating CD4^+^ T cells was measured. Corneal grafts were monitored for up to 8 weeks after transplant (*n* = 8 group), and Kaplan-Meier curves were plotted to evaluate graft survival. Representative slit lamp photography from either LR or LR are presented. BALB/C LR recipients were transplanted as above. Immediately after transplantation, allograft recipient mice received 2 × 10^4^ CMTMR-labeled iDC1 pretreated with IFN-γ or PBS (vehicle internal control) via SC injection. Two weeks after transplantation, mice were euthanized (*n* = 6 group). (**M** and **N**) CMTMR^–^ migratory CD103^+^ DC1 were sorted by FACS and analyzed by qPCR assay as above. (**O** and **P**) Intracellular expression of IFN-γ by Th1 (**O**) and FoxP3^+^CD25^+^ gated on CD3^+^CD4^+^ T cells (**P**) from the DLN of graft recipient mice was measured. (**Q**) Ex vivo Treg suppressive capacity on proliferating CD4^+^ T cells was measured. Additional mice were followed up (*n* = 8/group) for 8 weeks with slit lamp examination to evaluate graft opacity, and Kaplan-Meier curves were plotted to evaluate graft survival. Plots represent the mean (± SEM). *P < 0.05, **P < 0.01, ***P < 0.001, ****P < 0.0001 (2-tailed *t* test). All results are of 1 experimental set of animals with no repetitions.

Since alloreactive CD4^+^ T cells were observed in the DLN and considering inhibitory capacity of iDC1 ex vivo, we next investigated iDC1 inhibitory capacity in vivo. CMTMR-labeled iDC1 (2 × 10^4^) were SC transferred during transplantation (Figure 6A). We consistently observed: (a) CMTMR^–^ host CD103^+^ DC1 (gating strategy Supplemental Figure 6A) maintained their tolerogenic phenotype (Figure 6, B–H), (b) fewer alloreactive CD4^+^ T cells (Figure 6I, sorting strategy Supplemental Figure 6, B and C), (c) increased functional Tregs (Figure 6, J and K, sorting strategy Supplemental Figure 6, B and D), and (d) enhanced allograft survival (Figure 6L). The adoptive transfer of Treg (from recipients receiving iDC1) into newly transplanted HR recipients resulted in increased graft survival (Supplemental Figure 6, E and F). In contrast, LR recipients receiving iDC1 pretreated with IFN-γ (Figure 6M) showed (a) enhanced immunostimulatory phenotype in CD103^+^ DC1 (Figure 6N), (b) higher alloreactive CD4^+^ T cells (Figure 6O), (c) impaired Treg functionality (Figure 6, P and Q), and (d) allograft failure (Supplemental Figure 6, G and H). These data indicate that early intervention with iDC1 inhibits Th1 immunity while promoting Treg-mediated tolerance and graft survival in HR recipients.

### CD103^+^ DC1 modulate CD11b^+^ DC2 in the DLN but not in the graft site.

**Figure 7:**
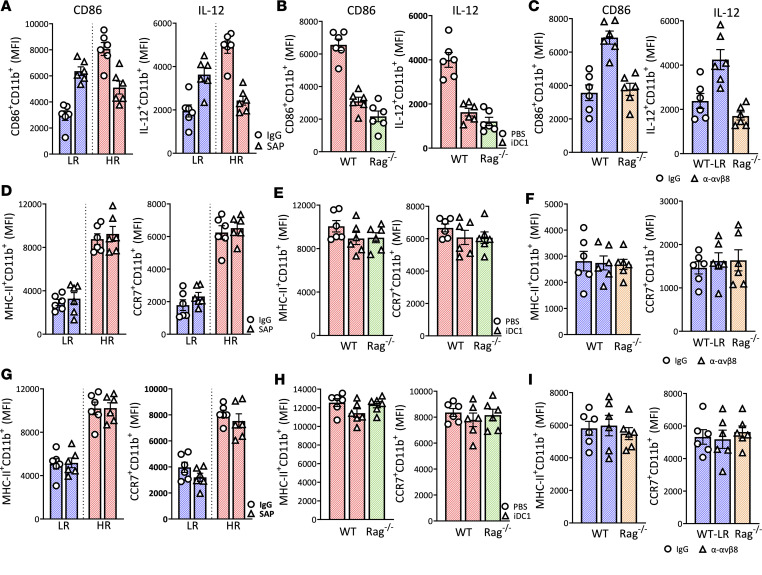
The effect of migratory CD103^+^ DC1 in modulating CD11b^+^ DC2 in the DLN and graft site. CD11b^+^ DC2 were analyzed by flow cytometry (gating strategy for grafted cornea and conjunctiva shown in Supplemental Figure 1 and for DLN shown in Supplemental Figure 2). (**A**–**C**) Expression of CD86 and IL-12 in the DLN of LR and HR transplanted mice treated with either anti–M290-SAP or anti–IgG-SAP (**A**), HR WT treated with either iDC1 or PBS and RAG^–/–^ transplanted mice (**B**), and LR WT and RAG^–/–^ transplanted mice treated with ADWA-11 antibody or IgG control (**C**) are shown. (**D**–**I**) In parallel, flow cytometry was performed on single-cell suspensions from the grafted corneas (**D**–**F**) and conjunctivae (**G**–**I**) of the same mice as described in **A**–**C**. Each symbol (**A**–**I**) indicates an individual mouse. Plots represent the mean (± SEM). One-way ANOVA with Bonferroni correction was used. All results are of 1 experimental set of animals with no repetitions.

Since CD103^+^ DC1 from HR recipients enhanced iDC2 immunostimulatory phenotype, we evaluated the effect on their physiological counterparts. We performed flow cytometry to assess CD11b^+^ DC2 maturation markers (gating strategy for DLN Supplemental Figure 2E; cornea and conjunctiva Supplemental Figure 1E). We observed that the depletion of CD103^+^ DC1 resulted in increased expression of CD86 and IL-12 in the DLN of LR recipients and decreased expression in HR recipients (Figure 7A).

On comparing WT and RAG^–/–^ recipients treated with either iDC1 or ADWA-11, we observed that iDC1 treatment resulted in decreased expression of CD86 and IL-12 by CD11b^+^ DC2 in HR WT recipients but not in RAG^–/–^ (Figure 7B). Conversely, ADWA-11 treatment resulted in increased CD86 and IL-12 by CD11b^+^ DC2 in WT but not in RAG^–/–^ recipients (Figure 7C).

Next, we analyzed CD11b^+^ DC2 maturation profiled by MHC-II and CCR7 expression at the graft site. CD103^+^ DC1 depletion in LR or HR recipients showed no effect on CD11b^+^ DC2 in the grafted cornea (Figure 7D) or conjunctiva (Figure 7G). A similar observation occurred in WT and RAG^–/–^ recipients treated with iDC1 in the cornea (Figure 7E) and conjunctiva (Figure 7H). Likewise, no differences were observed in WT RAG^–/–^ recipients treated anti-αvβ8 in the cornea (Figure 7F) or conjunctiva (Figure 7I). Collectively, these data indicate that migratory CD103^+^ DC1 counter-regulate the functional phenotype of CD11b^+^ DC2, which is dependent on CD4^+^ T cell interaction.

## Discussion

In this study, we used well-established models of corneal transplantation: a LR model of rejection, where approximately 50% of mice develop spontaneous tolerance and indefinite graft survival without any immunosuppressive therapy, and a HR model of rejection where 100% of allografts fail due to a host Th1 immune–mediated response. We observed that host CD103^+^ DC1 infiltrate the graft and migrate to the DLN and, in each model, play a distinct and critical role in transplant immunity.

In sharp contrast with the tolerogenic phenotype of migratory CD103^+^ DC1 observed in LR recipients profiled by low CD80/86 and IL-12 expression levels and high expression levels of IL-10 and other regulatory markers such as αvβ8, ALDH2, and PD-L1, we show migratory CD103^+^ DC1 acquire an immunostimulatory phenotype in HR recipients, while their prototypical lineage markers (Irf8, Id2, Batf3, Xcr1, and cd370) remain unchanged. Using immunodeficient mice and an ex vivo approach, we uncovered that migratory CD103^+^ DC1 undergo reprogramming in response to IFN-γ, resulting in upregulation of CD80/86 and IL-12, and downregulation of IL-10, αvβ8, and other immunoregulatory markers. In LR recipients, migratory CD103^+^ DC1 modulate the immunogenic activity of their migratory CD11b^+^ DC2 counterparts via IL-10, inhibit Th1 cells through the PD-L1/PD-1 axis, and promote Treg-mediated tolerance and graft survival.

In HR recipients, CD103^+^CD1 have a deleterious effect on the transplant immunity and graft survival. We observed that migratory CD103^+^ DC1 do not directly sensitize Th1 cells in HR recipients but instead increase the immunogenicity of migratory CD11b^+^ DC2 counterparts and prevent exhaustion of Th1 cells; both these changes are likely linked to elevated expression of IL-12 by CD103^+^ DC1. Finally, the adoptive transfer of tolerogenic iDC1 counteracts CD11b^+^ DC2 immunostimulatory capacity, suppresses CD4^+^ Th1 immune activation, and promotes Treg-mediated tolerance and graft survival.

C57BL/6 recipients display strong donor-specific delayed hypersensitivity and immediate allograft failure (36), which is a limitation of this study. Several strategies to silence transcription factors, such as IRF8 (27, 37, 38), Nfil3 (39), ID2 (40), Nr4a3 (41), or BATF3 (27, 42, 43), are effective for studying the function of CD103^+^ DC1 in C57BL/6 background mice; however, they are not suitable for our transplantation model in BALB/C recipients. To circumvent this intrinsic limitation, we used the immunotoxin M290-SAP, which induces apoptosis of CD103-expressing cells (44, 45). A single dose of M290-SAP is safe, depletes CD103^+^ DC1, and does not affect Treg populations; mice remain fully immunocompetent (20). We also used ADWA-11 antibodies to block αvβ8-mediated activation of TGF-β1 by CD103^+^ DC1 (29, 32–34), a critical step for the induction of tolerance (30, 46–48). Lastly, we used ex vivo expanded CD103^+^ DC1, a subset we previously defined as iDC1 (20). iDC1 resemble their physiological counterparts based on phenotypic markers, transcription factors, and functional capabilities (49, 50). iDC1 are not inherently tolerogenic, but stimulation with low doses of LPS (51), TGF-β1 (33, 34, 52), and ultraviolet-irradiated apoptotic corneal cells render them tolerogenic.

We observed that host CD103^+^ DC1 infiltrate the donor graft, resulting in increased frequencies in the graft site, similar to those observed in tumors (27, 43). Maximum infiltration of CD103^+^ DC1 was observed at 2 weeks after transplantation, consistent with the time that these cells take for differentiating from BM progenitors (20, 50). Graft-infiltrated CD103^+^ DC1 underwent maturation and migration to the DLN, which aligns with prior studies showing that expression of MHC-II and CCR7 is critical for the migration of CD103^+^ DC1 (27, 43, 50). The fundamental finding that migratory CD103^+^ DC1 (independent of their lineage markers) display differential functional phenotypes in LR compared with HR recipients aligns with the current paradigm of the hierarchical stratification of DC, which establishes that these cells adapt their phenotype to different environments independent of their signature profile (27, 29, 31, 53). We have previously shown that a CD103^+^ DC1 tolerogenic phenotype is associated with maturation after phagocytosis of apoptotic cells in the graft site (20). We found that these cells acquire an immunostimulatory phenotype upon sensing IFN-γ, which aligns with previous studies by Garris et al. (26) and Maier et al. (27), showing a pivotal role of IFN-γ as a main driver of induction into an immunostimulatory phenotype on tumoral-derived DC.

Antigen cross-presentation is the primary pathway of Th1 sensitization, and the involvement of passenger CD103^+^ DC1 in eliciting CD8^+^ Th1 responses in solid organ transplantation is very well established (31, 54). In murine models of corneal transplantation, only the central donor tissue is transplanted, and this region lacks CD103^+^ DC1 (20), which refutes the notion of passenger CD103^+^ DC1. Moreover, CD8^+^ Th1 cells’ involvement in corneal transplant immunity has already been discarded (55). We observed that migratory CD103^+^ DC1 capacity to sensitize CD4^+^ T cells was negligible, and it we primarily associated with their high expression of PD-L1; this aligns with the paradigm to protect themselves from killing cytotoxic T-lymphocytes (56). Migratory CD103^+^ DC1 also failed to inhibit IFN-γ^+^CD4^+^ T cells in HR recipients, the opposite of which was observed in LR recipients (20). We related this weak inhibition with low IL-10, which limits their immunosuppressive capacity (as described by Iberg et al.; ref. 31), and high IL-12, which subverts exhaustion of activated T cells (57, 58).

The critical role of CD11b^+^ DC2 in inducing Th2 and Th17 immunity is well established (59); however, in the setting of corneal transplantation, these cells induce CD4^+^ Th1-mediated immune response (18, 19, 60, 61). Our data show that, in LR recipients, migratory CD103^+^ DC1 counterregulate the immunogenic capacity of CD11b^+^ DC2 primarily mediated by IL-10, as observed by Lai et al., in a *Mycobacterium tuberculosis* model (62). In contrast, IL-12 secreted by migratory CD103^+^ DC1 enhanced CD11b^+^ DC2 immunogenic capacity in HR recipients. In contrast to Th2 polarization, CD11b^+^ DC2–mediated Th1 polarization mainly depends on IL-12 (63, 64), which explains the skewed CD11b^+^ DC2–mediated Th1 immunity following corneal transplantation.

We also observed that Treg stability and function appeared significantly impaired in the DLN of HR recipients, which was associated with the downregulation of immunoregulatory markers expressed by migratory CD103^+^ DC1. As observed in the gastrointestinal system, Treg-mediated tolerance in corneal allografts is mediated by peripherally induced Tregs (61, 65), and these cells are primarily generated by CD103^+^ DC1 over other DC subsets (26, 36, 66–68). Herein, we observed that depletion of CD103^+^ DC1 resulted in impaired Treg-mediated tolerance and graft failure, in line with previous work by Esterházy et al. (29), who reported that loss of the transcription factor IRF8 expressed by DC leads to loss of Treg induction. Moreover, we observed that iDC1-induced Treg-mediated tolerance prolonged the graft survival even in HR recipients, consistent with our previous work showing that the adoptive transfer of Treg from LR recipients into HR recipients promotes long-term graft survival (61, 65). Mechanistically, we associate this process with the expression of αvβ8 integrin in CD103^+^ DC1, which is critical for generating peripheral Tregs (32–34). Despite αvβ8 expression in tissue-resident Tregs, the lack of Treg-specific expression of integrin β8 (conditional *Itgb8*-KO Tregs) does not impair Treg stability at rest in the lymphoid tissue and does not affect Treg-mediated tolerance (66). Therefore, we delineated a role of αvβ8 integrin in transplant immunity in line with previous observations reported in the gut (32–34) and in tumor models (67).

In this study, we show that migratory CD103^+^ DC primarily serve as a compensatory mechanism to regulate excessive CD11b^+^ DC2–mediated Th1 CD4^+^ T cell activation; however, their acquisition of an immunostimulatory profile confirms their inherent protective role against foreign antigens (54, 68–70). Understanding this hierarchy between both migratory subsets is critically relevant for designing more specific therapeutic approaches for transplantation, where immune-mediated rejection is not only dependent on response of various DC subsets but also on the interaction between host and donor DC populations.

## Methods

### Sex as a biological variable.

Sex was not considered as a biological variable. Both male and female mice were used for all in vivo experiments.

### Animals.

Eight-week-old male and female BALB/c and C57BL/6 mice (Charles River Laboratories) and BALB/c Rag^–/–^ knock-out mice (The Jackson Laboratory) were used for experiments. All animals were housed in a pathogen-free vivarium at the Schepens Eye Research Institute Animal Facility and treated in compliance with the Association for Research in Vision and Ophthalmology Statement for the Use of Animals in Ophthalmic and Visual Research.

### Orthotopic corneal transplantation.

Mice were anesthetized with an i.p. injection of 120 mg/kg ketamine and 20 mg/kg xylazine for surgical procedures. For the LR model, grafts were placed in healthy corneas (Supplemental Figure 1A). For the HR model, corneal neovascularization was induced by placing 3 interrupted sutures (11–0 nylon, Sharpoint; Vanguard) in the paracentral intrastromal cornea extending over 120° of the corneal circumference for 2 weeks (Supplemental Figure 1B). After 2 weeks, the sutures were removed. Corneal transplantation was performed using C57BL/6 mice as allogeneic donors, BALB/c as syngeneic donors, and BALB/c or BALB/c Rag^–/–^ mice as graft recipients, as described previously (71–73). Briefly, the central cornea of donor mice was marked with a 2 mm diameter trephine and excised with Vannas scissors (Storz Instruments Company). Recipient graft beds were prepared by excising a 1.5 mm diameter area in the central cornea. Donor buttons were transplanted and secured with 8 interrupted 11–0 nylon sutures into host beds. Postoperatively, the eyelids were closed for 3 days with 8–0 nylon sutures. Corneal sutures were removed on day 7 after transplantation. Postoperatively, the eyelids were closed for 3 days with 8–0 nylon sutures. Corneal sutures were removed on day 7 after transplantation.

### In vivo depletion of CD103^+^ DC.

Recipient BALB/c or RAG^–/–^ mice received 2.0 mg/kg of M290-SAP (Advanced Targeting Systems Inc.) i.p. or the same dose of IgG conjugated to saporin (IgG-SAP). Previous studies have reported that M290-SAP delivered in this manner is cleared from circulation by day 3 after injection (74).

### I.p. injection of anti-αvβ8 antibodies.

Appropriate antibodies ADWA-11 (manufactured by ATUM and customized using UCSF-Sheppard Lab free license) for each group and isotype control antibodies were injected (10 mg/kg) on the day of the transplant and then once a week for the period of the experiments. The dose was based on previous publications (67).

### Cornea and conjunctiva digestion and lymph node cell preparation.

Excised corneas and conjunctivae were digested in RPMI media (Lonza Inc.) containing 2 mg/mL DNase I (Roche GmbH) and 0.5 mg/mL Collagenase D (Roche GmbH) for 60 minutes in 37°C and filtered through a 70 μm cell strainer. Ipsilateral DLNs (submandibular and cervical) and mesenteric lymph nodes were collected, and single-cell suspensions were prepared. Single-cell suspensions from the spleen were prepared, and the depletion of RBCs was performed with a RBC lysis buffer (Sigma-Aldrich).

### Flow cytometry.

Cell suspensions were incubated in PBS with zombie death/live (Supplemental Table 1) for 20 minutes before being washed with 10% FBS, incubated with an Fc receptor–blocking antibody (R&D Systems) for 20 minutes, and subsequently stained with the requisite antibodies or isotype controls (1:200; Supplemental Table 1). Stained cells were analyzed using an LSRII flow cytometer (BD Biosciences Inc.), and the acquired data were analyzed using FlowJo software (FlowJo LLC). For staining of IFN-γ, cells were stimulated with phorbol 12-myristate 13-acetate (PMA; 50 ng/mL; Sigma-Aldrich) and ionomycin (500 ng/mL; Sigma-Aldrich) in the presence of Golgistop (0.7 /100 μL media; BD Biosciences). Cells were fixed and permeabilized for intracellular staining with appropriate buffers (Thermo Fisher Scientific).

### Cell sorting.

CD45^+^Lin^–^CD11c^+^CD24^+^CD103^+^CD11b^–^ (DC1) and CD45^+^B220^–^CD11c^+^CD24^+^CD11b^+^CD103^–^ DCs (DC2) were isolated from the DLN of allograft recipient mice using BD Aria III FACS (BD Biosciences). CD4^+^CD25^–^ T cells were isolated from DLN of allografted recipient mice or the spleen of naive BALB/c mice by MACS using mouse T cell isolation kits (Miltenyi Biotec GmbH). CD4^+^CD25^+^ Tregs were isolated from DLN of allografted recipient mice and the lymph nodes and spleen of naive BALB/c mice by MACS using mouse Treg isolation kits (Miltenyi Biotec). The purity of sorted Tregs (frequencies of FoxP3^+^CD25^+^ cells) was > 90%, as determined by flow cytometry. The CD4^+^CD25^–^ fraction was used as conventional T cells (Tconv).

### Real time-PCR.

DCs from grafted corneas, conjunctiva, and DLN were sorted by FACS as described above and added directly to TRIZOL (Sigma-Aldrich) for RNA extraction. The RNA was extracted and purified using the RNeasy Micro kit (Qiagen Inc.). Total RNA was reverse transcribed using a Superscript III kit (Invitrogen Inc.). Real-time PCR was performed using TaqMan Universal PCR Mastermix (Applied Biosystems Inc.) and preformulated primers (Supplemental Table 1) with StepOne Real-Time PCR System (Applied Biosystems Inc.). The results were analyzed and normalized using GAPDH as an internal control.

### Immunofluorescence and confocal microscopy imaging.

Grafted corneas were harvested 2 weeks after transplantation and incubated in 20 mM EDTA (Sigma-Aldrich) at 37°C for 45 minutes to facilitate the epithelium removal. Subsequently, tissue was fixed in ice-cold acetone for 20 minutes, washed, and blocked with 3% BSA for 60 minutes. Next, corneas were incubated with Fc-blocking antibody (1:100, clone 2.4G2; BD Pharmingen) and FITC-CD103 and 594-CD11c antibodies (Supplemental Table 1) at 4°C overnight. Then, corneas were imaged using a TCS SP8 multiphoton microscope (Leica Microsystems Inc.), and images were processed using ImageJ software (NIH).

### In vivo generation of Tregs.

BALB/c-background RAG^–/–^ mice were transplanted with the allogeneic or syngeneic corneal tissue, and CD103^+^ DC were depleted in allograft recipients as described above. CD4^+^CD25^–^ T cells were sorted from the spleen and peripheral lymph nodes of BALB/c naive mice by depletion (Miltenyi Biotec) and adoptively transferred ( approximately 1 × 10^6^ cells/mouse) into graft recipients 3 days after transplant. The recipient mice were maintained in a specific pathogen–free environment. On day 21 after transplantation, the DLN were harvested, and the FoxP3 expression in CD4^+^CD25^–^ T cells was analyzed by flow cytometry.

### SC injection of Tregs.

Treg (1 × 10^5^ cells) isolated from the DLN of allograft-accepted recipient mice as described above, naive BALB/c mice, or CD4^+^CD25^−^ Tconv were suspended in 10 μL PBS and injected into the SC space of graft recipients using a 30 gauge metal needle and a 100 μL syringe (Hamilton Company Inc.) after corneal transplantation as we have done previously (72, 73). In our experiments, we injected Treg 3 days after the M290-SAP injection to allow clearance of M290-SAP (74).

### Evaluation of graft survival.

Transplanted corneas were examined weekly for 8 weeks in a masked fashion using a slit-lamp microscope. A standardized opacity grading system was used to define rejection (22, 75). Grafts with opacity scores higher than 2 for at least 2 consecutive weeks were considered immune rejected. Mice with postoperative complications such as synechiae or cataracts were excluded. Graft survival was assessed using Kaplan-Meier survival analysis.

### Ex vivo generation of iDC1.

Erythrocyte-lysed BM cells were prepared from femurs and tibiae of naive BALB/C mice as per the previous protocols (50, 76, 77). To induce iCD103^+^ DC (iDC1), 15 × 10^6^ BM cells were plated in sterile Petri dishes with 10 mL of RPMI 1640 (Lonza Inc.) based-complete medium (10 % FBS, 10 mM HEPES, 10 mM sulfate pyruvate, 10 mM nonessential amino-acids [NAA], 2 mM L-glutamine, 100 U/mL penicillin, 100 μg/mL streptomycin, and 50 μM 2-β-mercaptoethanol) (all from MilliporeSigma) in the presence of 300 ng/mL recombinant murine FLT3L (rmFLT3L; Peprotech Inc.) and 2 ng/mL of rmGM-CSF (Peprotech Inc.). After 5 days, 5 mL of complete medium was replaced to prevent cell death. On day 9, nonadherent cells were collected and counted, and 3 × 10^6^ cells were replated in 10 mL of complete medium supplemented with FLT3L and GM-CSF as above. On day 17, nonadherent cells were collected and filtered again. Next, cells were synchronized by serum starvation (in RPMI 1640 with 0.5% FBS) for 6 hours. Then 1 × 10^5^ cells were plated in an ultra–low-attachment polystyrene 96-well (round bottom) plate and stimulated for 12 hours with a low concentration of LPS (Sigma Aldrich) and murine recombinant active TGF-β1 (0.5 ng/mL, R&D Systems) and challenged with ultraviolet-irradiated apoptotic corneal cells from C57BL/6 mice. Depending on experiments, iDC1 were incubated with or without anti–PD-L1 (10 μg/mL, R&D systems) or IFN-γ (10 ng/mL, R&D systems). To generate iCD11b DC (iDC2), 2 × 10^6^ BM cells were plated in sterile Petri dishes with 10 mL of the same medium as above in the presence of 20ng/mL of rmGM-CSF (Peprotech Inc.) as previously described (78). On day 3, another 5 mL of medium containing 20 ng/mL of rmGM-CSF was added. Nonadherent iCD11b DC were harvested on day 7 and underwent starvation as above and plated in an ultra–low-attachment polystyrene 96-well (round bottom) plate (1 × 10^5^ cells) and stimulated with LPS (1 μg/mL) in the presence of ultraviolet-irradiated apoptotic corneal cells from C57BL/6 mice.

### In vitro Treg generation assay.

Migratory CD103^+^ DC1 (2 × 10^3^) or iDC1 (2 × 10^4^) sorted by FACS were washed twice and cocultured with syngeneic CD4^+^CD25^–^ T cells from naive mice (1:10 cell ratio) in 96-well (round bottom) plates in complete RPMI medium in the presence of 10 ng/mL of IL-2 (R&D Systems Inc.). Additionally, iDC1 (2 × 10^4^) were similarly cocultured with syngeneic CD4^+^CD25^–^ T cells from naive mice (1:10 cell ratio) in 96-well (round-bottom) plates in RPMI SFM in the presence of 10 ng/mL of IL-2 and recombinant latent TGF-β1 (0.5 ng/mL, R&D Systems Inc.). In addition, an anti-αvβ8 blocking antibody (50 μg/mL, ADWA11, Atum Inc.) was added. Cells were cultured for 5 days before analysis of Treg generation as assessed by flow cytometry.

### Treg suppression assay.

For proliferation assays, CD4^+^CD25^–^ T cells from the DLN of allograft recipient mice (responder cells) were cultured in plates with an anti-CD3 coated antibody (BioLegend). Briefly, plates were coated with 10 μg/mL of anti–mouse CD3 (BioLegend) in sterile PBS and incubated overnight at 4°C. Then, plates were washed 3 times with sterile PBS. Next, 2 × 10^6^/mL T cells were added and incubated for 2 days at 37°C. Subsequently, T cells were filtered, washed, and resuspended in PBS (20 × 10^6^/mL) at a final concentration of 1 μM of CFSE (Thermo Fisher Scientific) for 20 minutes at room temperature. The cells were washed in 10% FBS to neutralize extracellular CFSE and resuspended in the complete medium with anti-CD3/CD28 soluble antibody for 3 hours before coculture with iTregs. Treg were cocultured with CFSE-labeled T cells (1 × 10^5^) in a 96-well (round-bottom) plate in the presence of IL-2 (10 ng/mL) and anti-CD3/C28 soluble antibody for 4 days. The cells were harvested, and CFSE dilution was evaluated by flow cytometry. Percent suppression was calculated using the following formula: Percent suppression = ([Tconv proliferation without Tregs – Tconv proliferation with Tregs]/[Tconv proliferation without Tregs]) ×100.

### iDC1 suppression assay.

CD4^+^CD44^–^CD25^–^ T naive T cells were double stimulated using a CellXVivo Mouse Th1 Cell Differentiation Kit (R&D Systems). Briefly, naive CD4^+^ T cells (1 × 10^6^ cells/mL) were cultured in mouse Th1 differentiation media for 3 days following the manufacturer’s protocol. Culture plates were precoated with anti–mouse CD3 antibodies. After 3 days, T cells were harvested and stimulated by diluting 1:10 in fresh mouse Th1 differentiation media and cultured for 3 additional days in a flask. The flow cytometric analysis yielded a purity of approximately 85% IFN-γ ^hi^CD4^+^ T cells. To further characterize suppressive capacity, iDC1 (1 × 10^4^) that were previously incubated with or without anti–PD-L1 (10 μg/mL, R&D systems) or IFN-γ (10 ng/mL, R&D systems) were cocultured with Th1 cells (1 × 10^5^) for 24 hours. Then, cells were stimulated with PMA (50 ng/mL; Sigma-Aldrich) and ionomycin (500 ng/mL; Sigma-Aldrich) in the presence of Golgistop (0.7 μL/100 μL media; BD Biosciences) for 6 hours, and expression of IFN-γ ^+^ by CD4^+^ T cells was assessed by flow cytometry.

### MLR.

CD103^+^ DC1 were sorted by FACS from the migratory compartment of the DLN of HR recipient mice. BMDC-derived CD11b^+^ DC2 (iDC2) were generated with GM-CSF and stimulated with LPS. Naive CD4^+^CD25^–^ T cells were sorted by MACS from the spleen of naive BALB/C mice. Cells were cocultured for 3 days, and the intracellular expression of IFN-γ by CD4^+^ T cells was analyzed by flow cytometry and ELISA. A similar MLR was performed with iDC1 pretreated with or without either anti–PD-L1 (10 μg/mL, R&D systems) or IFN-γ (10 ng/mL, R&D systems) as a source of CD103^+^ DC1. CD4^–^CD11b^+^ DC2 were separated from CD103^+^ DC1 and analyzed by flow cytometry.

### MLR using a transwell system.

iDC2 and naive CD4^+^CD25^–^ T cells were cocultured in the lower and iDC1 anti–PD-L1 (10 μg/mL, R&D systems) or IFN-γ (10 ng/mL, R&D systems) were plated in the transwell insert. CD4^–^CD11b^+^ DC2 were separated from CD103^+^ DC1 and analyzed by flow cytometry. In addition, cell culture supernatants were collected, and IFN-γ, IL-12, and IL-10 levels were measured using the respective DuoSet Kit (R&D Systems).

### SC injection of iDC1 to allograft recipients.

iDC1 were washed 3 times in RPMI medium and labeled with 1 μM of either CellTracker Orange CMTMR (5-[-6]-[([4 chloromethyl] benzoyl) amino] tetramethylrhodamine). In total, 2 × 10^4^ cells were suspended in 10 μL PBS and injected SC to graft recipient mice using a 30-gauge metal needle and a 100 μL syringe (Hamilton Company) immediately after corneal transplantation. SC injection of PBS served as the control group.

### Statistics.

Kaplan-Meier survival analysis was used to determine graft survival, and the log-rank test was used to compare survival rates between groups. The 2-tailed Student’s *t* test was applied to compare quantitative data between 2 groups. One-way ANOVA with Bonferroni correction was used to determine whether there were any statistically significant differences between the means of 3 or more independent (unrelated) groups. The results are presented as mean ± SEM. *P* < 0.05 was considered statistically significant.

### Study approval.

All the experiments conducted for this study were approved by the IACUC of Schepens Eye Research Institute, and animals were treated in compliance with the Association for Research in Vision and Ophthalmology Statement for the Use of Animals in Ophthalmic and Visual Research.

### Data availability.

Values for all data points in graphs are reported in the Supporting Data Values file.

## Author contributions

Study concept and design were contributed by TB and RD. Acquisition, analysis, and interpretation of data were contributed by TB, HN, AM, RBS, MK, AN, HG, SIS, and RD. Drafting of the manuscript was contributed by TB and RBS. Critical revision of the manuscript for important intellectual content was contributed by RD. Statistical analyses were contributed by TB and SIS. Obtained funding was contributed by RD. Administrative, technical, or material support were contributed by TB. Study supervision was contributed by RD.

## Supplementary Material

Supplemental data

Supporting data values

## Version 1 09/05/2024

In-Press Preview

## Version 2 10/22/2024

Electronic publication
