# Supplementary material for: Acquired immunostimulatory phenotype of migratory CD103+ DCs promotes alloimmunity following corneal transplantation
Source: JCI Insight. 2024 Oct 22;9(20):e182469. doi: 10.1172/jci.insight.182469 (PMC11530131; doi:10.1172/jci.insight.182469)
Supplement: Supplemental data [file jciinsight-9-182469-s309.pdf]

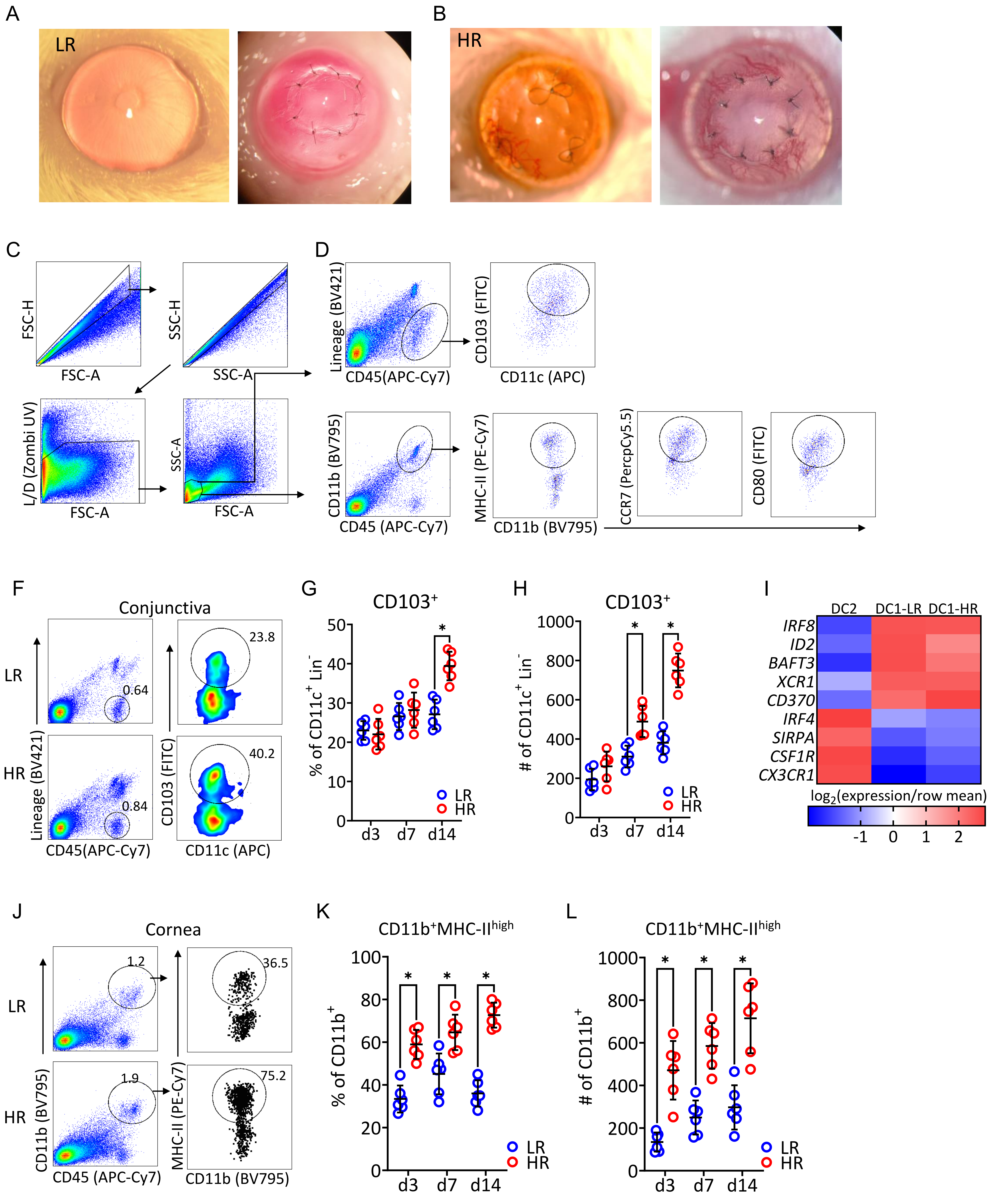

A
LR
B
HR
C
FSC-H
FSC-A
SSC-H
SSC-A
L/D (Zombi UV)
FSC-A
SSC-A
FSC-A
D
Lineage (BV421)
CD45(APC-Cy7)
CD103 (FITC)
CD11c (APC)
CD11b (BV795)
CD45 (APC-Cy7)
MHC-II (PE-Cy7)
CD11b (BV795)
CCR7 (PercpCy5.5)
CD80 (FITC)
F
Conjunctiva
LR
HR
0.64
0.84
23.8
40.2
Lineage (BV421)
CD45(APC-Cy7)
CD103 (FITC)
CD11c (APC)
G
CD103+
% of CD11c+ Lin-
d3 d7 d14
LR
HR
H
CD103+
# of CD11c+ Lin-
d3 d7 d14
LR
HR
I
DC2 DC1-LR DC1-HR
IRF8
ID2
BAFT3
XCR1
CD370
IRF4
SIRPA
CSF1R
CX3CR1
log2(expression/row mean)
-1 0 1 2
J
Cornea
LR
HR
1.2
1.9
36.5
75.2
CD11b (BV795)
CD45 (APC-Cy7)
MHC-II (PE-Cy7)
CD11b (BV795)
K
CD11b+MHC-IIhigh
% of CD11b+
d3 d7 d14
LR
HR
L
CD11b+MHC-IIhigh
# of CD11b+
d3 d7 d14
LR
HR

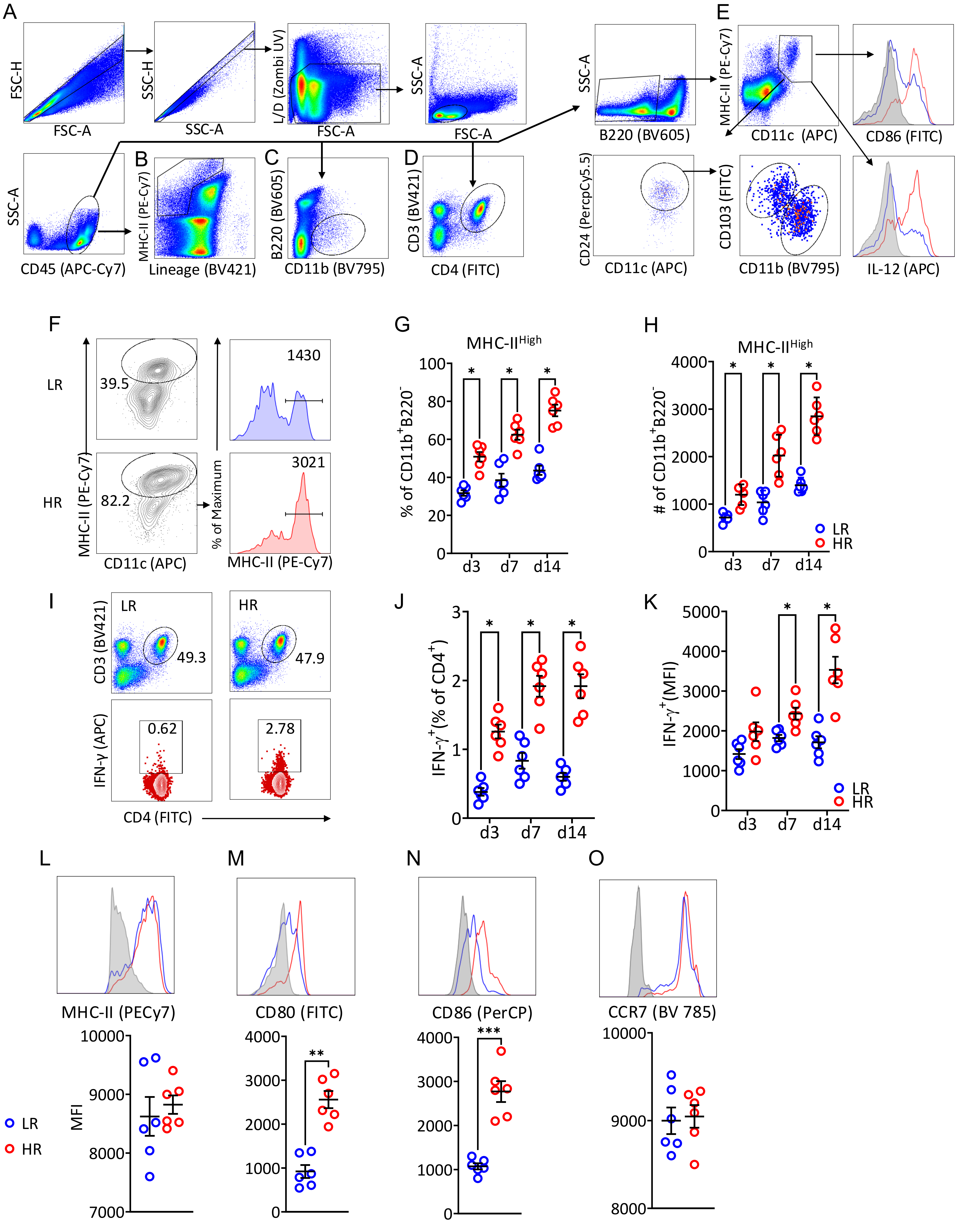

A
FSC-H
FSC-A
SSC-H
SSC-A
L/D (Zombi UV)
FSC-A
SSC-A
FSC-A
SSC-A
CD45 (APC-Cy7)
B
MHC-II (PE-Cy7)
Lineage (BV421)
C
B220 (BV605)
CD11b (BV795)
D
CD3 (BV421)
CD4 (FITC)
SSC-A
B220 (BV605)
E
MHC-II (PE-Cy7)
CD11c (APC)
CD86 (FITC)
CD24 (PercpCy5.5)
CD11c (APC)
CD103 (FITC)
CD11b (BV795)
IL-12 (APC)
F
LR
39.5
HR
82.2
MHC-II (PE-Cy7)
CD11c (APC)
1430
3021
% of Maximum
MHC-II (PE-Cy7)
G
MHC-II^High
% of CD11b+B220-
d3
d7
d14
H
MHC-II^High
# of CD11b+B220-
LR
HR
I
LR
HR
CD3 (BV421)
49.3
47.9
IFN-γ (APC)
0.62
2.78
CD4 (FITC)
J
IFN-γ+(% of CD4+)
K
IFN-γ+(MFI)
L
M
N
O
MHC-II (PECy7)
CD80 (FITC)
CD86 (PerCP)
CCR7 (BV 785)
MFI
LR
HR

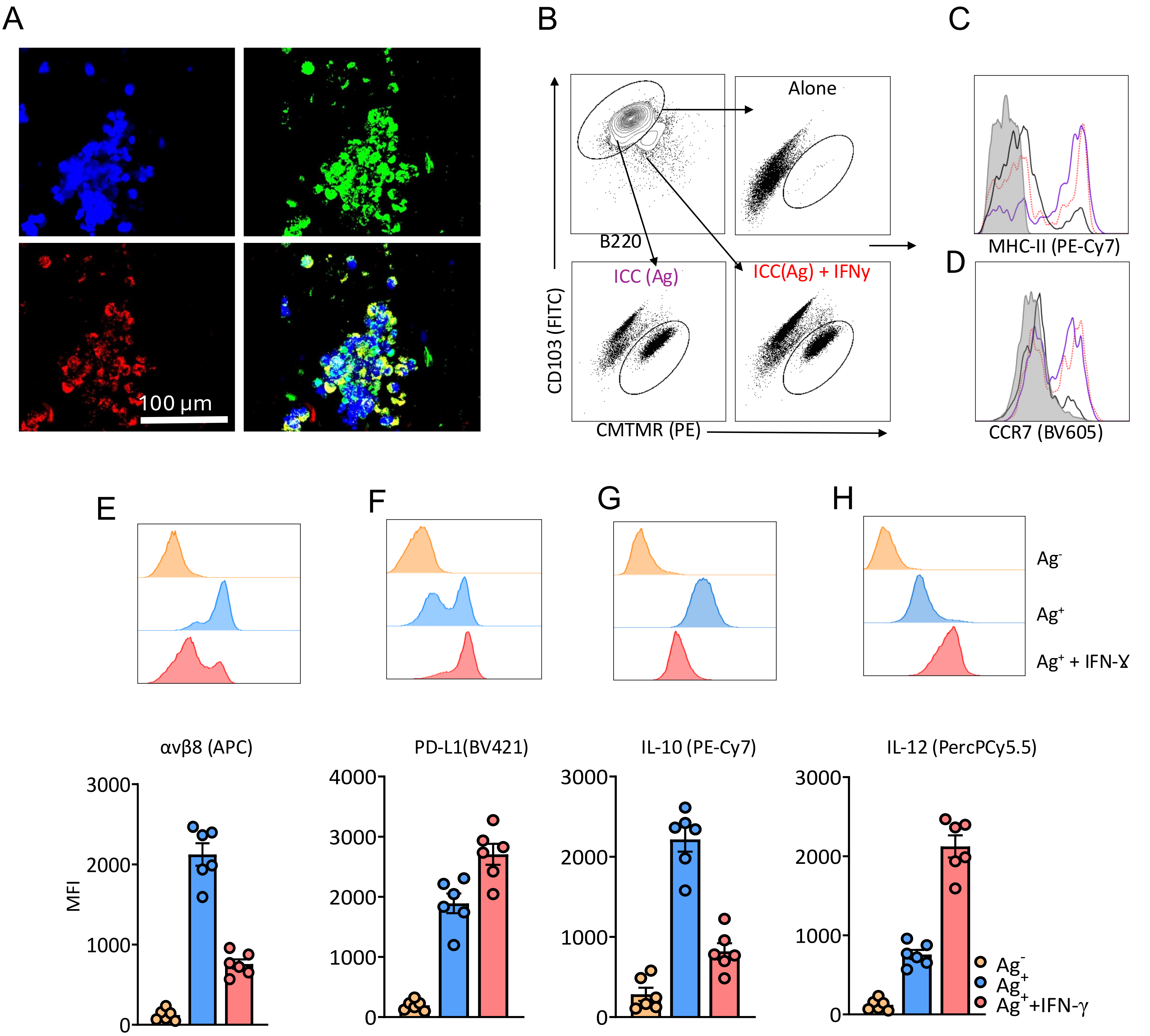

A
100 µm
B
Alone
B220
ICC (Ag)
ICC(Ag) + IFNy
CD103 (FITC)
CMTMR (PE)
C
MHC-II (PE-Cy7)
D
CCR7 (BV605)
E
F
G
H
Ag⁻
Ag⁺
Ag⁺ + IFN-ɣ
αvβ8 (APC)
PD-L1(BV421)
IL-10 (PE-Cy7)
IL-12 (PercPCy5.5)
MFI
3000
2000
1000
0
4000
Ag⁻
Ag⁺
Ag⁺+IFN-γ

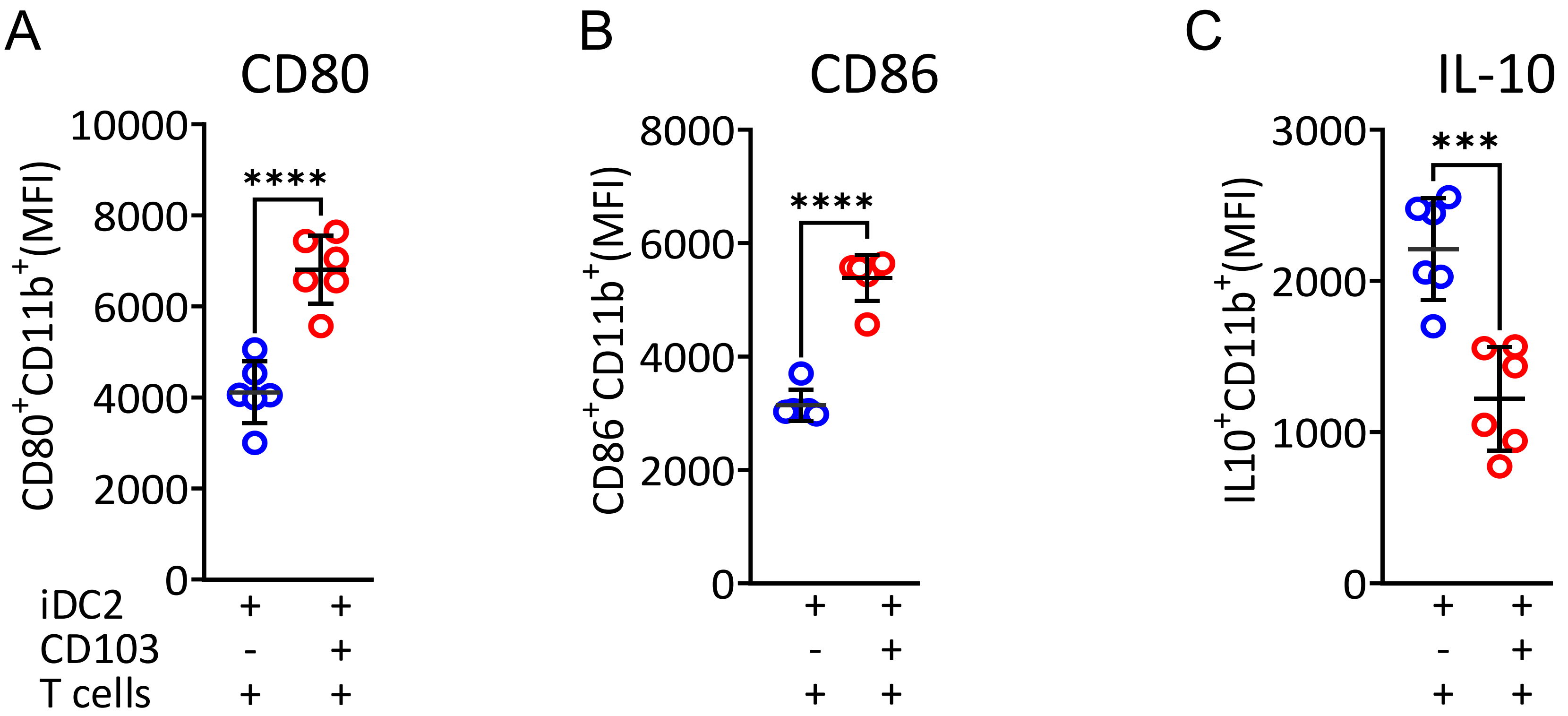
A
CD80
B
CD86
C
IL-10
CD80+CD11b+(MFI)
CD86+CD11b+(MFI)
IL10+CD11b+(MFI)
****
****
***
10000
8000
6000
4000
2000
0
3000
1000
iDC2
CD103
T cells
+ +
- +
+ +

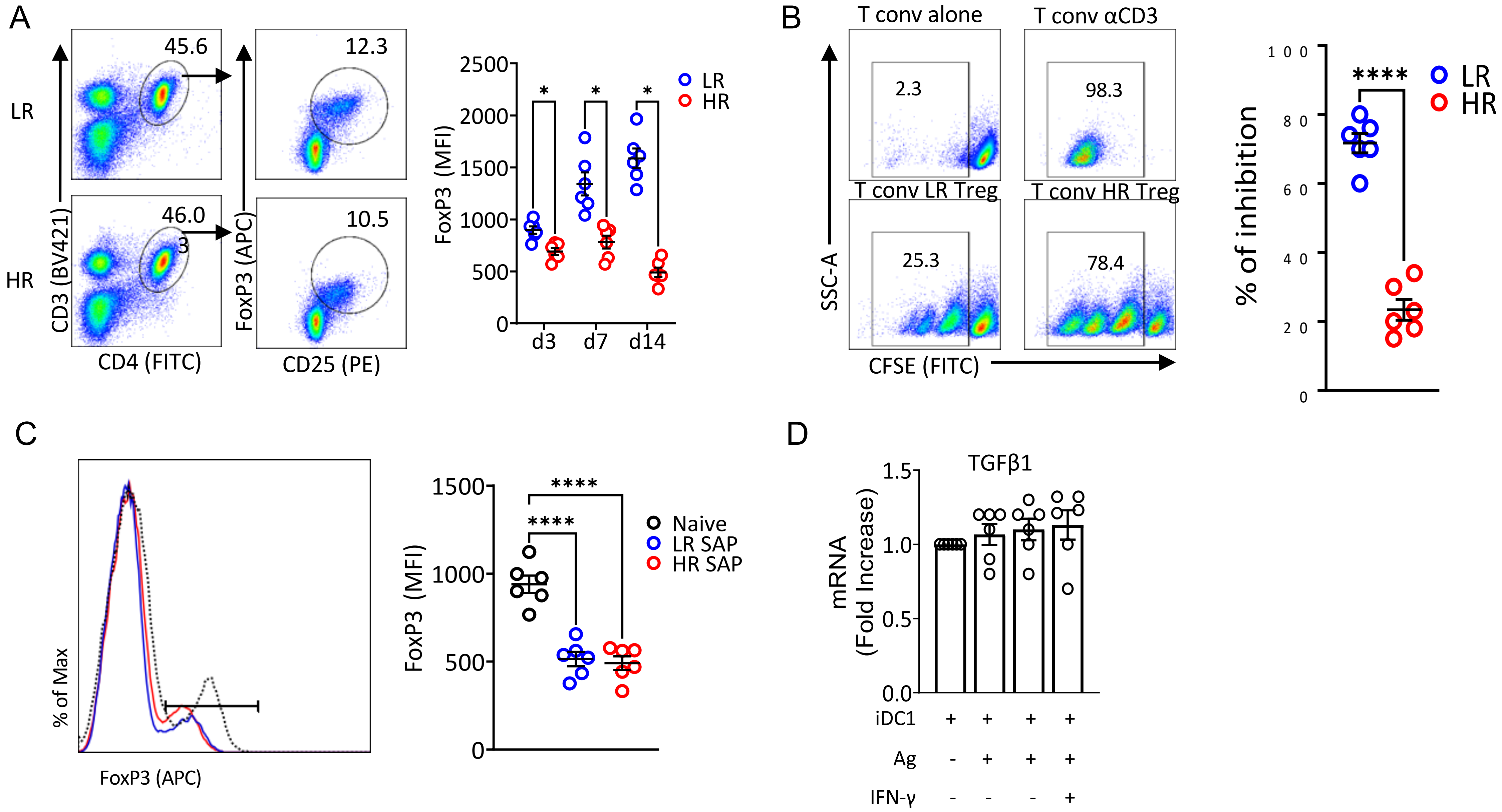
A
LR
HR
45.6
46.0
12.3
10.5
CD3 (BV421)
CD4 (FITC)
FoxP3 (APC)
CD25 (PE)
FoxP3 (MFI)
2500
2000
1500
1000
500
0
d3
d7
d14
*
LR
HR
B
T conv alone
T conv αCD3
T conv LR Treg
T conv HR Treg
2.3
98.3
25.3
78.4
SSC-A
CFSE (FITC)
% of inhibition
100
80
60
40
20
0
****
LR
HR
C
% of Max
FoxP3 (APC)
FoxP3 (MFI)
1500
1000
500
0
****
Naive
LR SAP
HR SAP
D
TGFβ1
mRNA
(Fold Increase)
1.5
1.0
0.5
0.0
iDC1
Ag
IFN-γ

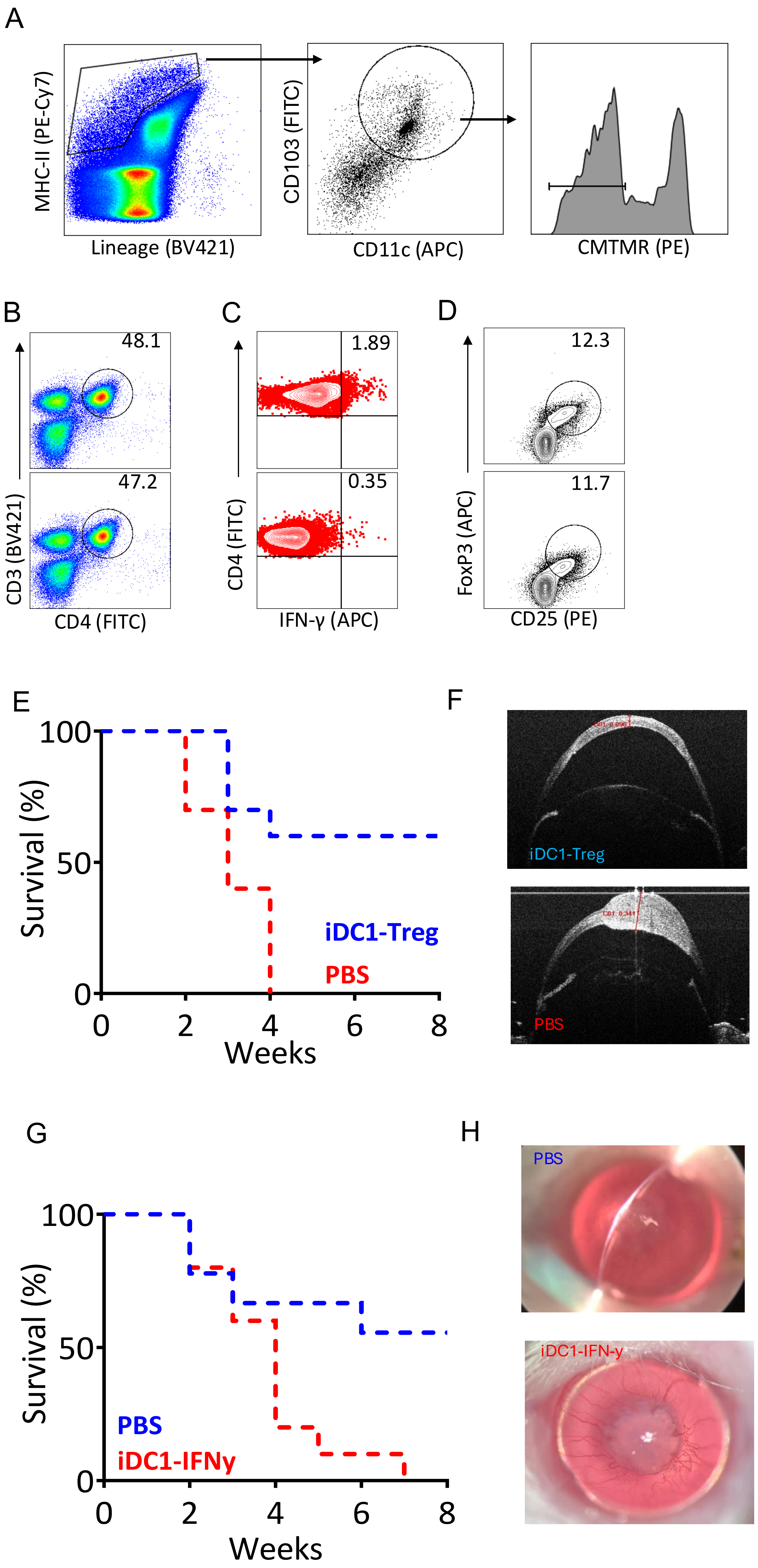

A
MHC-II (PE-Cy7)
Lineage (BV421)
CD103 (FITC)
CD11c (APC)
CMTMR (PE)
B
48.1
47.2
CD3 (BV421)
CD4 (FITC)
C
1.89
0.35
CD4 (FITC)
IFN-γ (APC)
D
12.3
11.7
FoxP3 (APC)
CD25 (PE)
E
100
50
0
Survival (%)
0
2
4
6
8
Weeks
iDC1-Treg
PBS
F
iDC1-Treg
PBS
G
100
50
0
Survival (%)
0
2
4
6
8
Weeks
PBS
iDC1-IFNγ
H
PBS
iDC1-IFN-γ

Supplemental Table 1: Antibodies, probes and software

**Flow Cytometry antibodies**				
CD45	1:200	A20	APC-CY7	Biolegend
CD11c	1:200	N418	APC	Thermofisher
Lineage	1:200	17A2; RB6-8C5; RA3-6B2; Ter-119; M1/70;	Pacific Blue	Biolegend
B-220	1:200	RA3-6B2	Pacific Blue	Biolegend
CD24	1:200	M1/69	BV510	Biolegend
CD103	1:200	2E7	BV785	Biolegend
CD103	1:200	2E7	FITC	Biolegend
CD11b	1:200	M1/70	PerCPCy5.5	Biolegend
CD11b	1:200	M1/70	BV785	Biolegend
IA/IE	1:2000	M5/114.15.2	PE-CY7	Biolegend
CD80	1:200	16-10A1	FITC	Biolegend
CD86	1:200	GL-1	PerCP-Cy5.5	Biolegend
CCR7	1:200	4B12	BV785	Biolegend
BTLA	1:200	6A6	PerCP-Cy5.5	Biolegend
αvβ8	1:100	416922	APC	Thermofisher
ALDH2	1:100	E4-D10	Unconjugated	Thermofisher
PD-L1	1:200	10F.9G2	BV785	Biolegend
PD-L1	1:200	10F.9G2	BV421	Biolegend

CD3	1:200	17A2	Pacific Blue	Biolegend
CD3	1:200	17A2	PE-CY7	Biolegend
CD4	1:200	RM4-5	FITC	Biolegend
CD4	1:200	RM4-5	APC-CY7	Biolegend
CD25	1:200	3C7	PE	Biolegend
FoxP3	1:100	FJK-16s	APC	Thermofisher
LAP-TGFβ1	1:100	TW7-16B4	FITC	Biolegend
IL-10	1:100	JES5-16E3	BV421	Biolegend
IL-12	1:100	C11.5	PerCP-Cy5.5	Biolegend
IFN-γ	1:100	XMG1.2	APC	Biolegend
Zombie UV	1:100		INDO-1 (Violet)	Biolegend
**Blocking antibodies, coating antibodies and agonist molecules**				
PD-L1	10 μg/mL	2341A	Unconjugated	R&D
IL-10	0.2 ng/mL	JES052A5	Unconjugated	R&D
IL-12	0.2 ng/mL	AF-419-NA	Unconjugated	R&D
αvβ8	50 μg/mL	ADWA11	Unconjugated	Atum Inc.
CD3	0.5 mg/ml	17A2	Purified	Biolegend
CD28	0.3 mg/ml	E18	Purified	Biolegend
IL-12	10 ng/mL	P43432	Recombinant	R&D systems
IFN-γ	10 ng/mL	NP_032363	Recombinant	R&D systems
**Immuno-fluorescence antibodies**				
CD103	1:100	2E7	FITC	Invitrogen

CD11c	1:200	2F1C10	CoraLite594	Proteintech

**FAM-MGB dye-labeled predesigned probes**			
**Name**	**Gene**	**Assay ID**	**Vendor**
IRF8	Irf8	Mm00492567_m1	Thermofisher
ID2	Id2	Mm00711781_m1	Thermofisher
BATF3	Batf3	Mm01318274_m1	Thermofisher
CLEC9A	Cd370	Mm00554956_m1	Thermofisher
IRF4	Irf4	Mm00516431_m1	Thermofisher
SIRPα	Sirpa	Mm00455928_m1	Thermofisher
CSF1R	Csf1r	Mm01266652_m1	Thermofisher
CX3CR1	Cx3cr1	Mm00438354_m1	Thermofisher
BTLA	Cd272	Mm00616981_m1	Thermofisher
Avβ8	Itbg8	Mm00623991_m1	Thermofisher
ALDH2	Aldh1a2	Mm00501306_m1	Thermofisher
PD-L1	Cd274	Mm00452054_m1	Thermofisher
IL-10	Il10	Mm01288386_m1	Thermofisher
IL-12	1I12a	Mm00434169_m1	Thermofisher
TGFβ1	Tgfb1	Mm03024053_m1	Thermofisher
GAPDH	Gapdh	Mm99999915_g1	Thermofisher
**ELISA Kits**			
Name			R&D
IL-12	Mouse IL-12 p70	M1270	R&D

IL-10	Mouse IL-10	M1000B	R&D
TGFβ1	Mouse TGF-beta 1	DY1679	R&D
IFN-γ	Mouse IFN-gamma	MIF00	R&D
**Software and algorithms**		**Vendor**	
GraphPad Prism 5.0		Graphpad	
ImageJ		NIH	
FlowJo		Tree Star	
Biorender		Biorender.com	
